# Statistics of high-level scene context

**DOI:** 10.3389/fpsyg.2013.00777

**Published:** 2013-10-29

**Authors:** Michelle R. Greene

**Affiliations:** Department of Computer Science, Stanford UniversityStanford, CA, USA

**Keywords:** context, scene, ensemble, bag of words, data mining, scene understanding

## Abstract

Context is critical for recognizing environments and for searching for objects within them: contextual associations have been shown to modulate reaction time and object recognition accuracy, as well as influence the distribution of eye movements and patterns of brain activations. However, we have not yet systematically quantified the relationships between objects and their scene environments. Here I seek to fill this gap by providing descriptive statistics of object-scene relationships. A total of 48, 167 objects were hand-labeled in 3499 scenes using the LabelMe tool (Russell et al., 2008). From these data, I computed a variety of descriptive statistics at three different levels of analysis: the ensemble statistics that describe the density and spatial distribution of unnamed “things” in the scene; the bag of words level where scenes are described by the list of objects contained within them; and the structural level where the spatial distribution and relationships between the objects are measured. The utility of each level of description for scene categorization was assessed through the use of linear classifiers, and the plausibility of each level for modeling human scene categorization is discussed. Of the three levels, ensemble statistics were found to be the most informative (per feature), and also best explained human patterns of categorization errors. Although a bag of words classifier had similar performance to human observers, it had a markedly different pattern of errors. However, certain objects are more useful than others, and ceiling classification performance could be achieved using only the 64 most informative objects. As object location tends not to vary as a function of category, structural information provided little additional information. Additionally, these data provide valuable information on natural scene redundancy that can be exploited for machine vision, and can help the visual cognition community to design experiments guided by statistics rather than intuition.

## Introduction

Imagine that you are attending a friend's housewarming party. Although you have never been in this house before, you are not surprised to find a coffee table next to a sofa in the living room, chairs surrounding the dining room table, or framed pictures hanging on the walls. As a considerate guest, you help with the cleanup afterwards; effortlessly finding the trash can under the sink for disposing the waste, and the dishwasher next to the cabinets to wash the dishes. Our interactions in the world are facilitated by virtue of the fact that objects are not randomly strewn about the world but follow some basic laws of where they may be located, how large they are, and what other objects will be found near them. Collectively, these regularities are known as *context*. While context appears to be crucial for human scene recognition and helpful for machine vision (see Bar, 2004; Oliva and Torralba, 2007 for reviews), contextual relations between scenes and their objects have not yet been systematically measured and cataloged.

### Why measure statistics of object context?

The last two decades have seen a growing literature on the statistics of natural images. Knowing about the input received by our visual systems allows for a better understanding of visual coding in the brain. We have a growing understanding of the statistical regularities of natural scenes at the level of basic features such as luminance, contrast, color and Fourier amplitude spectra, as well as the relations between edges and contours (Olshausen and Field, 1996; van Hateren and Ruderman, 1998; Fine and MacLeod, 2001; Geisler et al., 2001; Schwartz and Simoncelli, 2001; Golz and MacLeod, 2002; Torralba and Oliva, 2003; Howe and Purves, 2004). Mid-level regularities have been found for scene textures (Torralba and Oliva, 2003) as well as scene scale and depth (Ruderman, 1994; Torralba and Oliva, 2002). Higher-level statistical regularities, such as object location (Karklin and Lewicki, 2003), scene background to objects (Torralba and Sinha, 2001; Choi et al., 2010) and scene spatial structure (Schyns and Oliva, 1994) have also been measured. The importance of this work lies in the predictive power of image statistics for both behavior and neural responses (Rao et al., 2002; for reviews, see Simoncelli and Olshausen, 2001; Geisler, 2008). It has been hypothesized that the visual system exploits statistical redundancies in order to efficiently code a complex visual world (Attneave, 1954; Zetzsche et al., 1993; Barlow, 2001). Thus, knowing the statistical dependencies between objects and scenes can help us to understand the types of compressed visual codes that allow us to rapidly recognize our visual environments.

Despite a large and growing literature on the effects of context on object and scene recognition (Palmer, 1975; Friedman, 1979; Biederman et al., 1982; Boyce et al., 1989; De Graef et al., 1990; Henderson, 1992; Bar and Ullman, 1996; Hollingworth and Henderson, 1998; Henderson et al., 1999; Davenport and Potter, 2004; Eckstein et al., 2006; Neider and Zelinsky, 2006; Auckland et al., 2007; Becker et al., 2007; Davenport, 2007; Joubert et al., 2007; Võ and Henderson, 2009; Mack and Palmeri, 2010), there has yet to be a systematic quantification of scene-object relationships in the world. This is a critical step as recent work has found that principles of attention and perception learned from artificial laboratory stimuli have limited generalizability to real-world stimuli (Neider and Zelinsky, 2008; Wolfe et al., 2011a,b). Here I seek to fill this gap by providing both descriptive statistics of contextual relations and inferential statistics to show how much these types of context can contribute to scene categorization.

Suppose you wanted to know whether object recognition benefits from lawful scene context (e.g., Davenport and Potter, 2004). Traditionally, one would approach the problem by embedding the object of interest in normal a scene context (e.g., a “blender” in a *kitchen*), or an abnormal scene context (e.g., a “blender” in a *bathroom*), and then have human observers perform an object categorization task on both types of stimuli. Similarly, what if you wanted to study the degree to which an object evokes a particular scene context (e.g., Bar and Aminoff, 2003). Or perhaps you are interested in how scenes are formed from diagnostic objects (e.g., MacEvoy and Epstein, 2011). In each of these cases, how do you choose the object and scene contexts that you will use? How do we define diagnosticity for objects, and how do we measure it? Are all abnormal contexts equally bad? In each of these cases, these questions have been answered through introspection and intuition. The aim of this work is to provide baseline statistics of objects in scenes so that these types of questions can be answered with quantitative measures.

### Theories of object context

One of the first theories of object-scene context was known as *frame* or *schema* theory (Bartlet, 1932; Minsky, 1975; Friedman, 1979; Biederman, 1981). According to this theory, scene categories can be represented in a mental structure containing learned associations between the category and objects that are commonly found in it. For example, a *kitchen* schema might activate representations of objects such as “refrigerator,” “blender,” and “cutting board.”

Biederman et al. (1982) argued that there are five object-scene relationships that constitute well-formed visual scenes. Scenes must obey the laws of physics, with objects *supported* by a horizontal surface, and not occupying the same physical space (*interposition*). Furthermore, the objects themselves have a certain *likelihood* of being in a particular scene context, as well as some some probable *position* in it. Finally, every object is constrained to have a particular *size* relative to the other objects in the scene. The first two relationships describe physical constraints on the world, while the last three describe the semantic content of the scene. These authors found that violations in any of these relationships resulted in reaction time and accuracy deficits for object recognition within a scene, that multiple violations made performance worse, and that both types of relations (physical and semantic) disrupted scene and object processing to similar degrees.

Much of the experimental work on scene-object context has focused on the likelihood contextual relation, often referred to as *consistency*. It is generally accepted that a consistent object in a scene facilitates object and scene recognition (Palmer, 1975; Loftus and Mackworth, 1978; Boyce et al., 1989; De Graef et al., 1990; Bar and Ullman, 1996; Hollingworth and Henderson, 1998; Davenport and Potter, 2004; Eckstein et al., 2006; Becker et al., 2007; Joubert et al., 2007; Võ and Henderson, 2009, 2011; Mack and Palmeri, 2010). However, an open debate still exists over whether this facilitation is perceptually or cognitively based (Hollingworth and Henderson, 1998; Henderson and Hollingworth, 1999; Bar, 2004). The details of this argument are beyond the scope of this paper.

As there are no existing norms for object frequencies in scenes, it is often left to the intuitions of the experimenters to determine which objects are consistent or inconsistent in a scene category. Two salient exceptions include Friedman (1979), who obtained normative rankings by asking participants to brainstorm lists of objects that have various probabilities of being in a particular scene, and Henderson (1992), who provided a pilot experiment where the object-scene pairs were verified by an independent group of observers. However, in the absence of ground truth measurements of object frequency, the notion of object consistency seems to be better capturing object *plausibility* rather than object probability. For example, in Davenport and Potter (2004), a “sand castle” was chosen to be the consistent object for a *beach* scene. While sand castle is a very plausible object in a beach scene, most beaches are unlikely to have sand castles, making “sand castle” a plausible, but low-probability object. By measuring contextual statistics of objects and scenes, we can revisit the consistency effect with experiments reflecting real-world probabilities rather than intuitions.

There is also general agreement that context involves some form of learned associations extracted from interactions in the world. For example, in the phenomenon of contextual cueing (Chun and Jiang, 1998), observers' reaction times in repeated visual search displays become more efficient over the course of an experiment, suggesting that they implicitly learned the spatial layout of the displays. Brockmole and Henderson (2006) have shown that displaying a letter search array on a real-world scene and consistently pairing the target location with a location in the scene also produces contextual cueing. Furthermore, this result generalizes across different scenes in the same category (Brockmole and Võ, 2010), suggesting that object-scene relationships can be implicitly extracted and used for visual search. However, there remain a number of open questions: how strong are the contextual relations between objects and scenes? Does the strength of the relations differ among different types of scenes (e.g., indoor, natural landscapes, or urban environments)? Understanding and characterizing these relationships will allow the formulation of new experiments examining the extent to which human observers use various types of context for recognition, search and memory of complex natural scenes.

### Using context for rapid scene recognition

The mechanism behind the remarkable rapid scene categorization performance of human observers has been a long-standing mystery. How is a scene recognized as quickly as a single object when scenes contain many objects? Biederman (1981) outlined three paths by which an initial scene representation could be generated: (1) by recognizing a prominent object that is diagnostic of the scene's category; (2) by perceiving and recognizing global, scene-emergent features that were not defined; or (3) by recognizing and spatially integrating a few contextually related objects.

Although global, scene-specific features have been shown to be useful for scene categorization (Greene and Oliva, 2009), observers are able to also report a few objects after a brief glance at a scene (Fei-Fei et al., 2007). The first and third paths outlined by Biederman have been sparsely explored, as what counts as a “diagnostic” or “contextual” object is not immediately obvious. In this work, I operationalize these concepts so we may begin to test these hypotheses.

### Scope of the current work

In this paper, I introduce a large scene database whose objects and regions have been fully labeled using the LabelMe annotation tool (Russell et al., 2008). The fully labeled data contain names, sizes, locations, and 2D shapes for each object in each scene. In this work, I will provide descriptive statistics on these data at three levels of description: statistical ensembles, bag of words and structural. At the ensemble level, I will examine the overall object density and spatial distribution of unnamed objects and regions across the scene categories. The bag of words level of description uses the object labels to determine which objects occur in which scene categories without regard to the spatial distribution of these objects. The structural description will then examine the spatial relations among objects across scene categories. For each level of description, I will also describe how sufficient these statistics are for predicting scene categories through use of a linear classifier, and discuss how human observers may employ such strategies for rapid scene recognition.

## Methods

### Scene database

**Figure 1:**
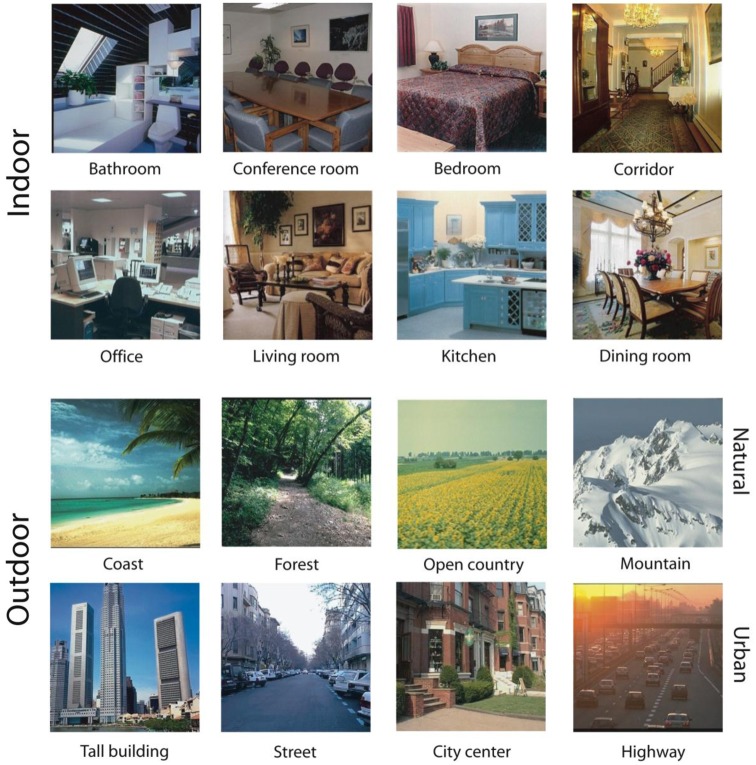
**Example images of each basic-level category**. The top two rows are from indoor scene categories and the bottom two are from outdoor scene categories. The top row of outdoor scenes is from natural landscape categories and the bottom row of outdoor scenes is from urban categories.

The main scene database consists of 3499 full-color scene photographs from 16 basic-level categories. Eight of the basic-level categories are indoor environments (*bathroom*, *bedroom, conference room, corridor, dining room, kitchen, living room*, and *office*). These images were downloaded from the web. The remaining scene categories were outdoor environments taken from Oliva and Torralba (2001), with four categories representing urban environments (*skyscrapers, street scenes, city centers*, and *highways*) and four categories representing natural environments (*coast, open country, mountain*, and *forest*). There were at least 94 images in each of the 16 basic-level categories. The images varied in size and were selected from a large lab database amassed from the web, personal photographs and books. See Figure 1 for example images from each basic-level category.

### Labeling procedure

The image database^1^ was hand segmented and labeled using the LabelMe Open Annotation Tool (http://labelme.csail.mit.edu, Russell et al., 2008) by four observers (including the author) over the period of several months. Observers were instructed to label all regions and objects in each image and affix the best basic-level name to each region as well as to label objects as individuals, size permitting (e.g., annotate each apple in a bowl of apples except in cases where apples were too small to create an accurate bounding region). It was decided in advance that objects that could be seen through windows would not be annotated, as these objects are not located in the given scene environment. Similarly, objects whose reflections appeared in mirrors were not annotated because this would artificially inflate the count of this object in the scene. Namable parts of objects that are not separable from the object (e.g., the leg of a chair, or headlight of a car) were not labeled. For the labelers, any visual, namable entity counted as an object, so items such as “fog,” “cloud,” or “sand” were considered objects. Although one typically thinks of “objects” as discrete entities that do not comprise the structure of a scene, regions vary in their “objectness.” In order to avoid idiosyncratic labeling strategies, all regions were considered. In cases of occlusion, labelers were instructed to interpolate object boundaries as to do otherwise would increase the count of this type of object. Statistical analysis on these annotations was performed in Matlab using the LabelMe toolbox (Russell et al., 2008).

### Cleaning the database

As the LabelMe interface accepts any label typed by an observer, the raw annotations contained numerous typos, misspellings and synonyms. Raw labels were hand-corrected to ameliorate these issues. These changes reduced the number of unique annotations from 1767 to 617. Misspelled items accounted for 21% of the changes (for example “automan” for “ottoman”). Plurals were changed to singular, accounting for 15% of the changes. Labels that were written at the subordinate level, including descriptions of position information (“side view of car” or “slated wooden panel”) were changed to the appropriate entry-level category. These accounted for 40% of the changes. Furthermore, items listed at the superordinate level were visually inspected and assigned to the appropriate entry-level category, accounting for 3% of the changes. For example, “art” was a label that referred to a “painting” in one image and a “sculpture” in another, and “island” could refer to either a landmass in water or counter space in the center of a kitchen. In cases where the entry-level category of an object was questionable, attempts were made to group objects by function. For example, “decoration” was chosen as an entry-level as all objects under this label served a common function (e.g., “decorative wall hanging” or “decorative fish”). Object labels that were synonyms according to WordNet (Miller, 1995) were unified under one label (for example, “couch” and “sofa”). Synonyms accounted for 16% of the changes. Labels that encompassed multiple objects (for example, “basket of magazines”) were included as the containing, or larger object only (e.g., “basket,” 2% of changes). Labels that referred to object parts that are not independent of the object whole (e.g., “chair leg” is a part that is not removable from a chair without the chair losing its function) were deleted. Parts that could refer to the whole object (e.g., “back of chair” for a chair that was occluded except for the back) were changed to the object's name. These accounted for 2% of the changes. Finally, there were 288 labels that were simply called “object.” These referred to a variety of small objects that could not be accurately identified from the small images, so the label has not been changed. There were a total of 21 deletions. The list of deletions can be found in Appendix C. A list of raw and final labels can be found in Appendices A and B, respectively.

### Auxiliary dataset

Although ~3500 images is a relatively large database and near the practical limit of what one can hand-annotate, a critical question for the utility of these statistics is the degree to which they generalize to the population of all real-world environments. Indeed, dataset bias is known to limit the knowledge gleaned from this type of inquiry (Torralba and Efros, 2011). In order to address this question, I compared the contextual statistics from the main database with a completely independent labeled database. As every database has independent bias, the extent to which statistics measured in one database can be successfully applied to another reflects the generalizability of the database.

**Figure A1:**
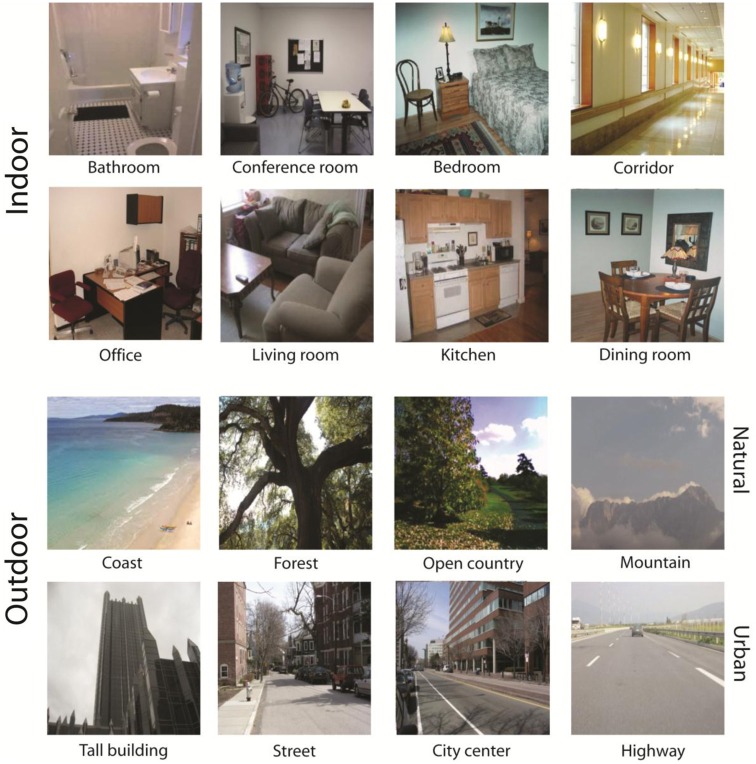
**Example images from the auxiliary database**.

I created an auxiliary set of images taken from the LabelMe database and annotated by unknown observers. The dataset consisted of 1220 images from the same 16 basic-level scene categories that had at least 85% label coverage. There were 100 images per category for *bathroom*, *kitchen*, *living room*, *city*, *street*, *coast*, and *forest*, and 14–59 in the others, as LabelMe does not have a sufficient number of fully labeled scenes for the other categories. These scenes were labeled by unknown observers without the rules used by the four observers who annotated the main set. As LabelMe allows users to upload their own photographs, this dataset differs from the main dataset in that the depicted environments are less idealized and stylized and seem to come from users snapping views of their own offices, kitchens and streets (see the Figure A1). This set was cleaned as described above. All analyses were repeated on this additional set, and all differences between the two databases are noted in Appendix D.

## Results

### General findings

#### Quality of annotations

How much of each image was labeled? Although labelers were instructed to label each pixel, some regions were too small to label, or may have been overlooked. Here I examined the percentage of image pixels assigned to a label. On average, 85.4% of an image's pixels were assigned to a label (standard deviation: 12.7%). Sixty one percent of images had more than 90% of its pixels labeled. By contrast, only 8.9% of images in the LabelMe database have this level of annotation (Russell et al., 2008) making the main database better suited to describing contextual statistics.

### Ensemble statistics

Ensemble statistics are statistical summaries of a group of objects, such as mean size (Ariely, 2001; Chong and Treisman, 2003, 2005), center of mass (Alvarez and Oliva, 2008), or mean orientation (Parkes et al., 2001). Although most work in this area has been on laboratory displays of simple shapes, human observers can estimate ensemble statistics over more complicated sets of features as well, such as the average emotion of a crowd of faces (Haberman and Whitney, 2007). Recent work in visual cognition has shown that the human visual system is adept at representing such ensembles both rapidly and outside the focus of attention (Ariely, 2001; Chong and Treisman, 2003, 2005; Alvarez and Oliva, 2008, 2009; for a review see Alvarez, 2011). Although the use of statistical ensembles has been posited as a potential mechanism for scene gist recognition (Haberman and Whitney, 2012), there has been little work on what statistical ensembles might exist in real-world images.

Here, I examined several summary statistics representing the general density and location of objects in scene categories. The utility of each measure for scene categorization is assessed both individually and as a group using a linear classifier.

#### Object density and variety

The first ensemble statistic is simply the density of labeled objects in each scene. The number of objects in a scene ranged from 1 to 88 (median: 11). Do all scene categories have a similar number of objects? To answer this question, I examined the number of objects per scene as a function of basic- and superordinate-level scene category labels. While human observers have no problems recognizing scenes with a variety of object densities (Potter, 1976; Wolfe et al., 2011a,b), classical visual search experiments show clear performance costs as the number of objects in a display increases (Biederman, 1988; Vickery et al., 2005). In order to better understand how the number of objects in a scene affects categorization performance in that scene, it is important to first understand how scenes vary in terms of object density.

**Figure 2:**
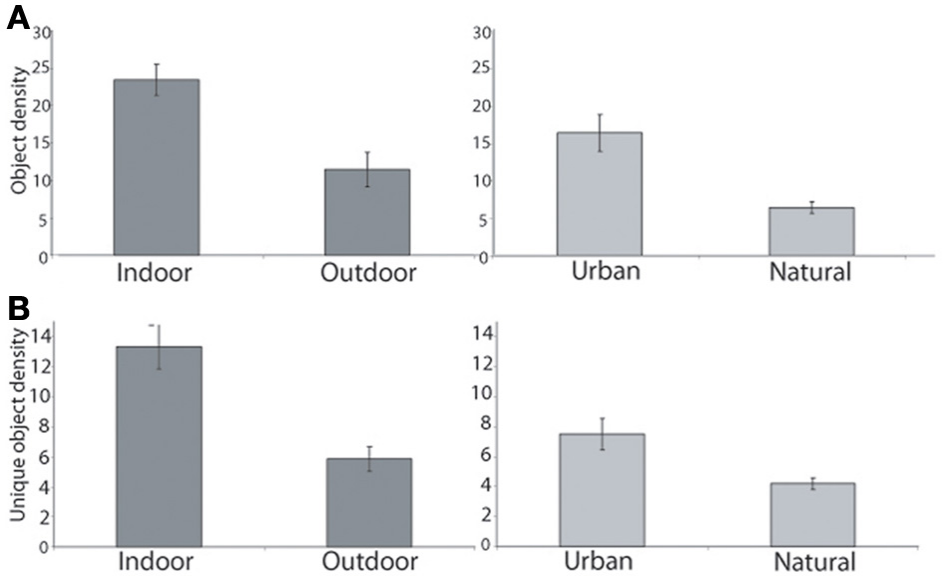
**(A)** Indoor scenes had more objects on average than outdoor scenes (left). Among the outdoor scenes, urban scenes had a greater number of objects than natural (right). **(B)** Indoor scenes had a greater number of unique labels in each scene than outdoor. Among outdoor categories, urban scenes had more unique objects than natural scenes. Error bars reflect ± 1 SEM.

**Figure 3:**
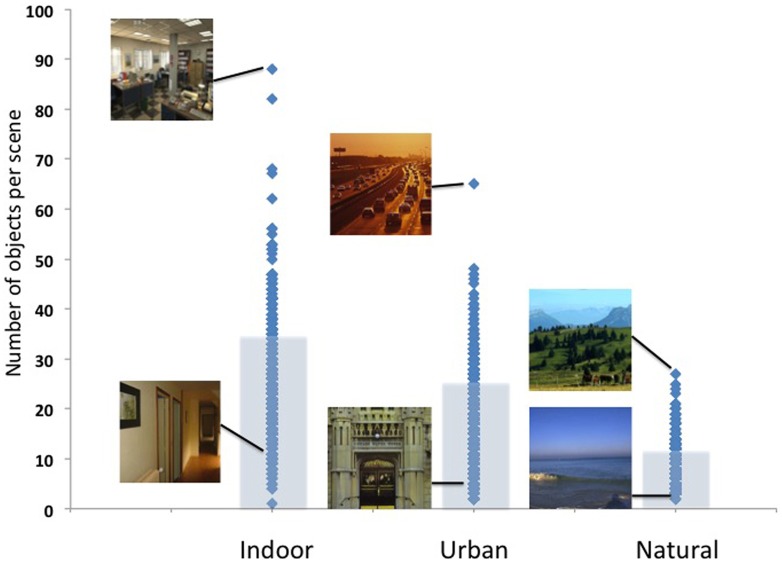
**Object density as a function of superordinate-level scene category (indoor, urban, and natural)**. Image examples of the most and least dense images are shown for illustration.

In this database, the mean object density ranged from 5.1 objects per *mountain* scene to 33.1 objects per *kitchen* (see Table 1A). As shown in Figures 2A, 3, indoor scenes had a significantly higher mean object density than outdoor scenes [23.45 and 11.44 objects per scene, respectively, *t*_(14)_ = 4.14, *p* < 0.01], and among the outdoor scenes, urban environments had a significantly higher average density than natural [16.43 vs. 6.46 objects per scene, respectively, *t*_(6)_ = 4.52, *p* < 0.01]. This indicates that the degree of human intervention in an environment results in more labeled regions and objects.

**Table 1:** **(A) The mean number of total labeled regions per scene for each of the basic-level scene categories; (B) the mean number of uniquely labeled regions per scene**.

	**Indoor**								**Urban**				**Natural**			
	**Bath**	**Bed**	**Conf.**	**Corr.**	**Dine**	**Kit' n.**	**Liv.**	**Off.**	**Tall**	**City**	**Strt.**	**High**	**Cst.**	**OpC**	**Mntn.**	**Frst.**
(A)	20.2	18.3	25.8	15.6	22.6	33.1	24.8	27.1	12.3	20.0	20.1	13.3	5.5	7.4	5.1	7.8
(B)	14.7	12.5	8.6	7.9	12.2	19.5	15.2	15.9	5.0	9.0	8.4	7.7	4.4	7.7	3.2	4.5

*Scene category abbreviations (left to right) are: bathroom, bedroom, conference room, corridor, dining room, kitchen, living room, office, tall building (skyscraper), city, street, highway, coast, open country, mountain and forest. This convention will be followed for all tables in this article*.

I also examined the number of unique objects in each scene: the larger this number, the greater the heterogeneity and possibly the complexity of the scene. In the database, the number of unique items in a scene ranged from 1 to 42 (median: 6). The number of unique regions in a scene varied from 19.5 in a *kitchen*, to 3.2 in *mountain* scenes (see Table 1B). As with total object density, there were more unique objects in indoor scenes when compared with outdoor scenes [13.3 and 5.7 unique items per scene, *t*_(14)_ = 4.78, *p* < 0.001], and among outdoor scene categories, more unique objects in urban scenes compared with natural scenes [7.55 and 3.93 unique items per scene, *t*_(6)_ = 3.76, *p* < 0.01, see Figure 2B). Manufactured environments therefore have both a greater number and greater variety of objects than natural environments.

#### Mean and variance of object size

Human observers are able to quickly and accurately compute the mean size of objects in laboratory displays (Ariely, 2001; Chong and Treisman, 2003, 2005). Are statistical properties of object size diagnostic of scene category? Although object size and density are related, it is important to consider that the two-dimensional labeling of a three-dimensional scene results in overlapping polygons. For example, a pillow on a bed will overlap with the bed, or a chair in front of a table with overlap with the table. Thus, mean object size is not trivially the inverse of object density.

For each scene, the size of each object was expressed as percent of total image area. In general, labeled regions were relatively small (the median mean object size was 17% of the total image area). There was considerable range in mean object size in our database, from a minuscule 0.05% of image area to a nearly all-encompassing 99.4%. Among basic-level categories, *living rooms* had the smallest mean object size (5% of image area) and *mountains* had the largest (43%), see Table 2. Predictably, indoor scenes had a smaller mean object size compared to outdoor scenes [7.5 vs. 24.4%, *t*_(14)_ = 4.94, *p* < 0.001]. Among the outdoor superordinate-level categories, natural scenes trended toward having a larger mean object size compared to urban [30.2 and 18.6%, respectively, *t*_(6)_ = 2.28, *p* = 0.063].

**Table 2:** **Mean (top) and standard deviation (bottom) of mean object size in percentage of total image area**.

**Indoor**								**Urban**				**Natural**			
**Bath**	**Bed**	**Conf.**	**Corr**	**Dine**	**Kiİn.**	**Liv.**	**Off.**	**Tall**	**City**	**Strt**	**High**	**Cst.**	**OpC**	**Mn tn.**	**Frst.**
5.7	6.9	10.3	14.0	7.1	5.4	5.0	5.3	24.9	17.4	20.0	12.0	23.9	25.2	42.9	29.0
2.7	3.3	5.5	7.4	3.8	3.7	2.1	2.7	15.4	11.6	9.5	6.5	11.6	12.1	15.3	22.4

Next, I examined object size variance across basic- and superordinate-level scene categories. For each scene, the size variance of all objects in the scene was computed. For each basic-level category, I computed the mean of object size variance, finding that *living rooms* had the smallest variance of object size, and *mountains* had the largest. Overall, indoor scenes had smaller variance of mean object size compared to outdoor [*t*_(14)_ = 5.84, *p* < 0.001], but no reliable difference was found between natural and urban scenes [*t*_(6)_ = 1.07, n.s.].

#### Center of mass

The previous ensemble statistics have shown us that, relative to outdoor environments, indoor scenes have a higher density of objects, and lower variance of object size. However, these do not tell us anything about where these objects are located in the scene. Previous work has shown that human observers are sensitive to the center of mass of a group of objects and can accurately compute this location even when attention is diverted elsewhere (Alvarez and Oliva, 2008). Are there robust differences in the locations of objects in different basic- and superordinate-level scene categories?

For each scene, the center of each object was computed as (xMax-xMin, yMax-yMin) of the polygon vertices. The center of mass for the scene was then computed as the mean of these values, weighted by the size of the object (as computed above). As expected, there was a strong tendency for the objects to center along the vertical axis (basic-level category centroids were located between 46 and 53% of total horizontal extent), indicating that objects were located with equal probabilities in the left and right sides of a scene. I observed a certain degree of diversity in position in the vertical axis, with basic-level category centroids occupying 35–75% of the vertical axis. This makes sense, as vertical location is a possible cue for scene depth. In particular, outdoor environments had a higher center of mass in the image plane (65% of vertical axis) than indoor environments [47% of vertical axis, *t*_(14)_ = 4.09, *p* < 0.01], reflecting the presence of objects such as skyscrapers, buildings and sky. However, no systematic difference was found between the natural and urban outdoor scenes [*t*_(6)_ < 1, n.s.]. Therefore, vertical center of object mass may contain diagnostic information for scene category.

#### Object spacing regularity

The center of object mass tells us about the general location of objects in an image, but this statistic does not tell us about the spacing of these objects. Objects that cluster together can be perceptually grouped (Gestalt law of proximity), and may have functional relations in a scene. Do scene categories vary in their object spacing regularity?

For each scene, pairwise distances between each of the objects in the scene were computed, using the (x,y) locations of each object's center of mass. Then for each scene, I computed the variability of object spacing as the standard deviation of distances, normalized by the mean distance between each object. Normalizing by the mean allows us to compare images that were not the same size. A low degree of variability indicates a very regular, grid-like spacing of objects while a high degree of variability suggests a more clustered spatial layout. While basic-level categories varied in their degrees of spacing variability, no systematic differences were found between indoor and outdoor scenes [t_(14)_ = 1.04, n.s.] nor between the natural and urban outdoor scenes [*t*_(6)_ = 1.12, n.s.]. This seems to be partially due to the fact that indoor scene categories were themselves quite variable: *bathroom* scenes displayed the highest degree of object spacing regularity of all 16 categories while *living rooms* displayed the lowest.

#### Scene classification with an ensemble statistics model

To what extent do these ensemble statistics provide information about the basic- and superordinate-level scene categories? To examine this question, I expressed each image in the database according to its object density, unique object density, mean object size, object size variance, center of mass and variability of object spacing. Using a support vector machine (SVM) classifier [linear kernel, using LIBSVM, Chang and Lin (2011)], I tested basic- and superordinate-level scene categorization. Each image was separately used as a test image after training with the remaining 3498 images in the database. This procedure was the same for all SVM analyses in this manuscript. LIBSVM uses a one-against-one multi-class classification, with all parameters remaining the same for each classification task. All default parameters for LIBSVM were employed. For the superordinate-level categorization task, the classifier achieved an accuracy of 91% correct for natural, 63.4% for urban and 76.5% for indoor scenes (overall AUC: 0.83). This overall level of performance is well above the chance level of 33% (binomial test, all *p* < 0.0001).

**Figure 4:**
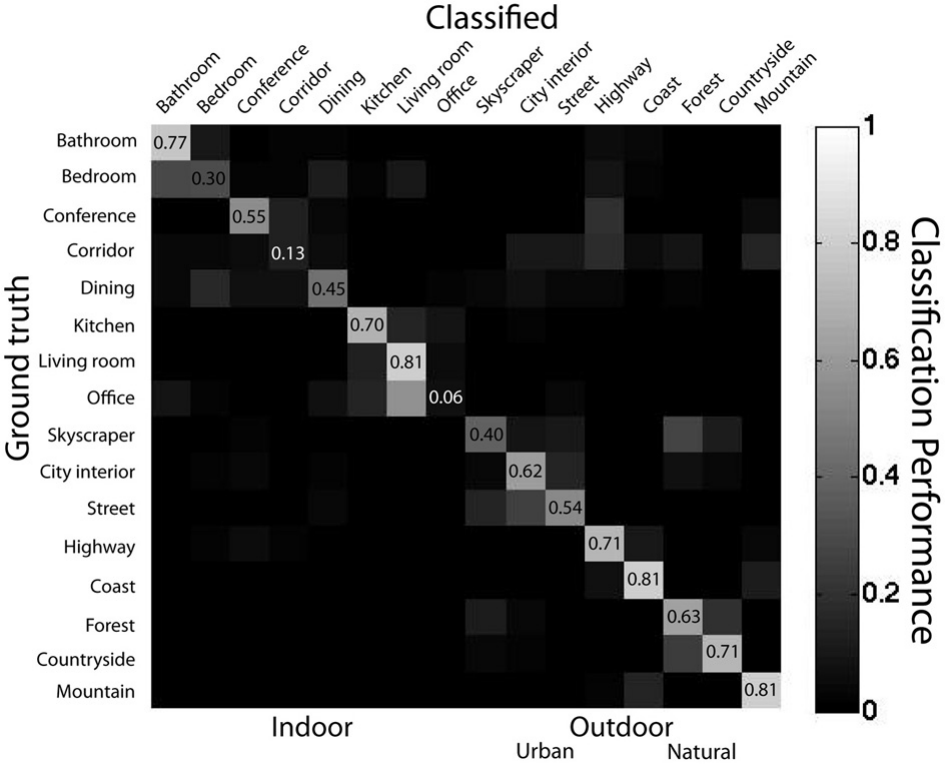
**Confusion matrix for linear SVM classifier representing ensemble statistics from 16 scene categories**. Light colors along diagonal show correct classifications while light colors on the off-diagonals represent misclassifications. Data from main database using SVM with linear kernel using leave-one-out cross validation.

For the basic-level categorization task, mean performance was 61% correct (AUC = 0.77), well above the chance level of 6.25%. Performance on each basic-level category ranged from 6% for *offices* to 81% for *living rooms*. Binomial tests on the performance of each basic-level category indicated that all categories except for *office* were classified above chance (*p* < 0.01). Basic-level classification performance did not differ significantly between outdoor and indoor scene categories [65 and 47% correct, respectively, *t*_(14)_ = 1.7, *p* = 0.10], nor did basic-level categorization performance differ among natural and urban scene categories [73 and 55% correct, respectively, *t*_(6)_ = 1.8, *p* = 0.12]. Incorrectly classified *offices* were frequently classified as *living rooms* (67% of mistakes), *kitchens* (13%), or *bathrooms* (10%), see Figure 4 for full confusion matrix.

What was the nature of the misclassifications? Twenty seven percent of misclassified scenes were misclassified across the indoor-outdoor superordinate distinction (i.e., a scene was indoor and was classified as one of the outdoor categories). This pattern is unlike human scene categorization, where mistakes are nearly always within the same superordinate-level category (Renninger and Malik, 2004; Kadar and Ben-Shahar, 2012). Although this classifier has remarkably high performance given its simplicity, the pattern of performance suggests that human observers use different or additional information for performing rapid scene categorization.

How does each of the ensemble statistics contribute to classification performance? To address this question, I performed the same SVM analysis as described above, using only one ensemble statistic at a time. Basic-level categorization performance was above chance for each of the ensemble statistics (binomial test, all *p* < 0.001), and ranged from 10.2% for center of mass to 31.6% for spacing regularity. A one-way ANOVA on the accuracy of each classifier revealed significant differences in performance (*p* < 0.001), suggesting that certain ensemble statistics are more useful than others for categorization. As shown in section **Object Spacing Regularity**, the regularity of object spacing did not differ reliably among superordinate level scene categories, even though it has the highest basic-level categorization performance when tested alone, indicating that this feature carries information about a scene's basic-level category, but not superordinate-level category.

In order to understand how the dimensionality of these features affects classification performance, I ran classifiers trained and tested on each combination of 2–6 ensemble statistics. Classification performance grew linearly in this range (slope: 8.3% per feature, *r*^2^ = 0.98). Extrapolating, ceiling performance could be expected with 11 ensemble features.

How well do ensemble statistics from the main database generalize to the auxiliary database? I trained a linear SVM on ensemble statistics from the main database, and tested categorization performance on the auxiliary database. Above-chance performance of this classifier indicates shared information because bias between the two databases should not be strongly correlated. Indeed, basic-level categorization performance for a model trained on the main database and tested on the auxiliary set was 17% (AUC = 0.52), significantly above chance level (binomial test, *p* < 0.001), indicating that ensemble statistics measured from one database contain information about the pattern of ensemble statistics in an independent database.

How does classifier performance compare to human performance on a rapid scene categorization task? Here, I compared the classifier to the human categorization data of Kadar and Ben-Shahar (2012) who tested observers on 12 of the 16 categories in the current database. In their experiment, participants were briefly shown two images and were then asked to determine whether the images were in the same category. Images were presented for 27–1000 ms and followed by a 1/f noise mask. The authors published confusion matrices for the scene categories averaged over presentation time. Overall, sensitivity of the ensemble statistics classifier was lower than that of the human observers [mean A' = 0.65 for classifier, 0.85 for participants *t*_(22)_ = 7.7, *p* < 0.0001]. When comparing the confusion matrices of the classifier to those of the human observers, I found that although the patterns of classifier confusion were not well correlated with human error patterns at the basic-level (*r* = 0.04), error patterns were quite similar when averaged over superordinate-level categories (*r* = 0.79). Therefore, the ensemble classifier can predict human performance at rapid scene categorization at a coarse level, adding support for the plausibility of such a coding scheme as a mechanism for scene gist perception.

Together, these analyses show that simple ensemble statistics, such as the number and location of nameless objects, are sufficient for above-chance scene categorization at both the basic and superordinate levels, and that the pattern of performance mimics human categorization performance at a coarse level.

#### Ensemble statistics discussion

In this section, I have described real-world images in terms of very simple statistics that express the quantity and coarse spatial distributions of “things” in a scene. These are of interest because they are rapidly computed by human observers on laboratory displays (Parkes et al., 2001; Chong and Treisman, 2005; Haberman and Whitney, 2007) and may explain aspects visual representations outside the fovea (Balas et al., 2009) or outside the focus of attention (Alvarez and Oliva, 2008, 2009).

Descriptively, these results show that statistical ensembles vary considerably with the degree of human manufacturing of an environment. In particular, indoor scenes have more total objects, a greater variety of objects, and a smaller average object size when compared to outdoor scenes. This same trend holds for urban scenes when compared to natural scenes, as urban scenes have a higher degree of manufacture. Spatially, indoor scenes had a lower center of mass compared to outdoor scenes. There are two reasons for this. Outdoor scenes have more objects further off the ground than indoor scenes (“sky,” “cloud,” “skyscraper,” “bird,” “tree”). Also, outdoor scenes also have a larger mean depth than indoor scenes (Torralba and Oliva, 2002). Objects receding in depth tend to be located higher in the x-y image plane, leading to a higher center of mass for outdoor scenes. Therefore, although scenes are treated as a single class in the literature, this result suggests that scenes are a heterogeneous set of entities, leaving open the possibility that different environments may be processed differently by the visual system.

Through the use of a linear classifier, I have shown that such simple statistics carry sufficient information to categorize a scene at both the basic- and superordinate-levels significantly above chance, demonstrating for the first time that ensemble statistics could be a plausible mechanism for scene gist recognition in human observers. Although this classifier had lower performance than the human observers from Kadar and Ben-Shahar (2012), the patterns of errors made by this model were similar to those made by the human observers when averaged over superordinate-level categories, suggesting that human observers may build an initial scene representation using ensemble-like features. Of course, the majority of work on ensemble statistics has been on very sparse laboratory displays. It remains to be seen whether observers can accurately report statistical information from complex, real-world images.

### Bag of words models

The statistical ensemble model considered all annotations to be nameless “things.” However, the identity of these “things” is critical to scene identity. In linguistics, models that consider statistical patterns of word use independent of syntactical relations (so-called “bag of words” models) have been successful in document classification and spam detection (Deerwester et al., 1990; Blei et al., 2003). In computer vision, growing bodies of models perform similar operations on visual “words” given by interest-point or object detectors (Sivic and Zisserman, 2003; Fei-Fei and Perona, 2005). Visual bag of words models have been very successful for scene classification in recent years (Bosch et al., 2006; Lazebnik et al., 2006; Li et al., 2010).

In the model considered here, a scene is represented as a list of the objects contained in it. Measures such as object frequency (overall as well as conditioned on scene category) and mutual information between objects and scenes will be employed while still ignoring the spatial relations existing between these objects and regions. As before, I will examine the fidelity of a bag of words model for predicting basic- and superordinate-level scene categories through the use of a linear classifier, and evaluate proposed schemes by which human rapid scene categorization might occur via object recognition.

#### Overall object frequency

**Figure 5:**
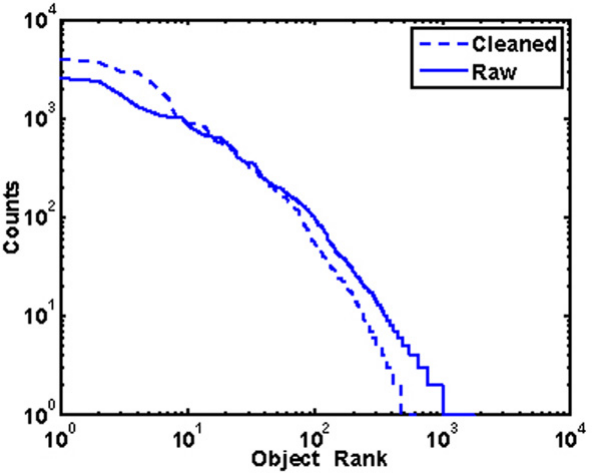
**Object frequency is inversely proportional to frequency rank**. This pattern does not strongly depend on how the database was cleaned.

Which objects are most common in the world? Just as certain words are more common than others in written text (“the” is more common than “aardvark”), certain objects appear in the world with greater frequency than others. Each of the 617 uniquely labeled regions in the database appeared between 1 and 3994 times in 1–2312 images. Nearly a quarter of the labels (22.7%) appeared only once in the database while eight objects (0.23%) appeared more than 1000 times. Overall, the frequency of objects in the database is inversely proportional to the frequency rank of the object, a relationship known in the linguistics literature as Zipf's law (Li, 1992; see Figure 5).

The 10 most common objects are listed in Table 3 where I list both the total counts for an object (right column), and the number of scenes that contain at least one instance of that object (middle column). It should be noted that these counts represent a lower bound for the number of objects in the scenes. In scenes where several exemplars of a small object were grouped together, but too small to individuate (e.g., “apples” in a “bowl,” or “books” on a “shelf”), it was typical for annotators to list these as a group using the plural.

**Table 3:** **The 10 most common objects in the database**.

**Object name**	**Nb scenes (%)**	**Total counts**
Sky	2312 (66)	2393
Tree	1377 (39)	3680
Building	1139 (33)	3994
Mountain	963 (28)	1615
Road	871 (25)	1064
Window	821 (23)	2981
Car	635 (18)	2943
Door	488 (14)	901
Ceiling	484 (14)	513
Plant	465 (13)	859

*The middle column shows the number of scenes containing at least one exemplar of this object. The percentage of scenes with this object is shown in parentheses. The right column shows the total number of these objects in the database*.

#### Object frequency

What are the most frequent objects in each scene category? Knowing object frequency will allow us to find out how sensitive human observers are to these frequencies, and thus better understand the role of expectation in scene perception.

Table 4 shows the 10 most frequent objects in each basic-level scene category. It is of note that there are relatively large differences between basic-level scene categories in terms of the frequency of the most typical objects: while “sofa” is an intuitively important object for *living rooms*, it was present in only 86% of living room scenes, while “faucet” was labeled in over 99% of *bathrooms*.

**Table 4:** **The 10 most frequent objects in each basic-level category along with the proportion of scenes in each category that contain at least one exemplar of that object**.

**Indoor**								**Urban**				**Natural**			
**Bath**	**Bed**	**Conf.**	**Corr.**	**Dine**	**Kit' İn.**	**Liv.**	**Off.**	**Tall**	**City**	**Strt.**	**High**	**Cst.**	**OpC**	**Mntn.**	**Frst.**
Faucet	Bed	Chair	Wall	Chair	Cabinet	Sofa	Desk	Building	Building	Building	Road	Sky	Sky	Mountain	Tree
0.90	0.88	0.99	0.99	0.98	0.96	0.86	0.94	0.95	0.94	1.00	1.00	0.99	0.96	1.00	0.96
Towel	Pillow	Table	Floor	Table	Counter	Pillow	Chair	Sky	Window	Road	Sky	Ocean	Tree	Sky	Sky
0.79	0.81	0.95	0.95	0.96	0.94	0.83	0.89	0.94	0.89	0.96	0.95	0.99	0.67	0.96	0.51
Sink	Lamp	Ceiling	Ceiling	Window	Window	Table	Monitor	Skyscraper	Door	Sky	Car	Mountain	Field	Tree	Bush
0.77	0.76	0.61	0.91	0.70	0.67	0.80	0.76	0.80	0.72	0.96	0.78	0.44	0.61	0.30	0.31
Bath	Ceiling	Light	Door	Bouquet	Sink	Lamp	Window	Tree	Road	Car	Tree	Sand	Mountain	River	Rock
0.69	0.73	0.61	0.76	0.66	0.63	0.68	0.70	0.52	064	0.89	0.77	0.42	0.51	0.09	0.25
Mirror	Window	Window	Light	Ceiling	Plant	Plant	Keyboard	Road	Sky	Sidewalk	Sign	Rock	River	Snow	River
0.68	0.70	0.43	0.49	0.56	0.62	0.67	0.67	0.22	0.58	0.77	0.54	0.33	0.24	0.09	0.23
Floor	Curtain	Painting	Window	Painting	Stove	Painting	Book	River	Sidewalk	Person	Fence	Boat	Hill	Rock	Grass
0.63	0.64	0.35	0.29	0.55	0.62	0.64	0.62	0.19	0.52	0.54	0.36	0.17	0.22	0.08	0.17
Toilet	Painting	Plant	Column	Light	Faucet	Window	Lamp	Car	Car	Tree	Mountain	Building	Building	Road	Ground
0.60	0.64	0.33	0.21	0.53	0.60	0.64	0.41	0.12	0.37	0.45	0.34	0.15	0.17	0.07	0.14
Window	Nightstand	Door	Painting	Curtain	Ceiling	Ceiling	Paper	Sidewalk	Tree	Van	Median	Sun	Bush	Ground	Mountain
0.59	0.62	0.30	0.21	0.41	0.55	0.61	0.41	0.11	0.32	0.27	0.33	0.14	0.14	0.06	0.13
Bottle	Bouquet	Cabinet	Table	Door	Floor	Armchair	Plant	Streetlight	Person	Streetlight	Building	Tree	Path	Mtn pass	Path
0.45	0.40	0.27	0.15	0.41	0.55	0.51	0.38	0.09	0.31	0.25	0.32	0.14	0.10	0.05	0.13
Wall	Dresser	Pr.	Plant	Lamp	Bowl	Rug	Ceiling	Antenna	Plant	Sign	Streetlight	Cloud	Rock	Person	Land
0.43	0.39	screen	0.13	0.36	0.54	0.47	0.37	0.08	0.25	0.23	0.28	0.11	0.10	0.05	0.12
		0.19													

**Figure 6:**
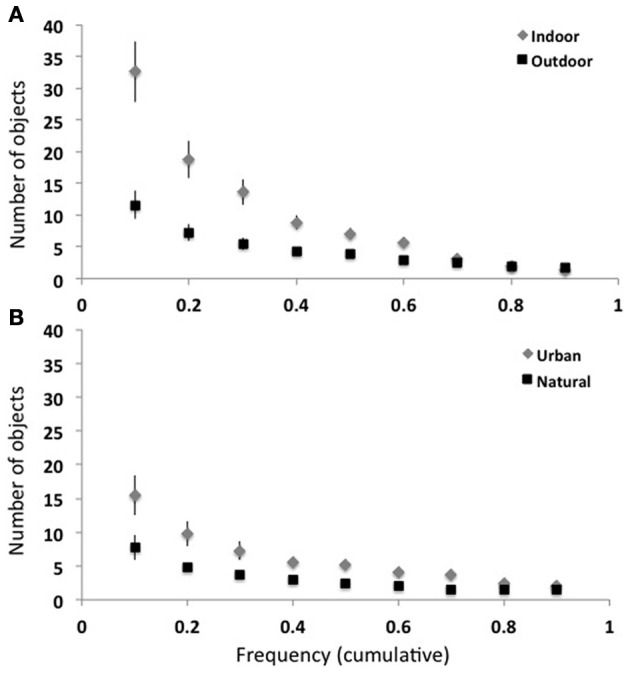
**Object frequency at various thresholds. (A)** Indoor scenes have more frequent objects than outdoor scenes at frequencies ≤0.5 (left). **(B)** Urban scene categories trend toward having more frequent objects than natural scene categories.

What is the overall frequency-rank relationship for each of the 16 scene categories? For each basic-level scene category, I computed the number of objects that were present in at least half of the images. Indoor scenes had a greater number of frequent objects compared to outdoor scenes [7.1 vs. 3.9 objects, *t*_(14)_ = 3.1, *p* < 0.01]. Among the outdoor scenes, urban scenes had a greater number of frequent objects compared to natural [5.3 vs. 2.5, *t*_(6)_ = 4.0, *p* < 0.01]. Again, this pattern shows that the degree of human manufacture affects the distribution of object frequencies. To probe at a finer level of detail, I computed the number of objects at frequency levels between 0.1 and 0.9. Figure 6 shows the average of outdoor and indoor scenes (top, A) and the average of natural and urban scenes (bottom, B). *T*-tests performed at each threshold level showed that no statistical difference exists between the number of objects in outdoor and indoor scenes for frequency thresholds above 0.5 (Bonferroni corrected), suggesting that although indoor scenes have more objects than outdoor scenes, all scenes have similar numbers of very frequent objects. Among the outdoor scene categories, natural and urban scenes did not reliably differ.

#### Object diagnosticity

How important is an object to scene identity? Important objects may frequently occur in scenes, but not all frequent objects provide information about scene category. For example, some objects, such as “tree” can occur in many environments, while other objects such as “toilet” can only occur in a specific context, such as a *bathroom*. To formalize this notion, I introduce *diagnosticity*, which is the probability of a scene belonging to a particular scene category conditioned on the presence of a particular object [p(scene|object)]. Although “chair” is a frequent object in *dining rooms*, chairs are not diagnostic of dining rooms because they are also found in *bedrooms, conference rooms, offices*, etc. Similarly, there may be objects that are diagnostic that are not frequent, and these might reflect object-scene pairs that have been used in the object consistency literature (recall the example of the “sand castle” on the *beach*). This measure is of particular interest as some models of human rapid scene categorization posit that categorization can be mediated through the recognition of one or more diagnostic objects (Friedman, 1979; Biederman, 1981).

Diagnosticity was measured for every object and scene category in the database. However, this metric over-represents rare objects. As nearly one quarter of labeled objects occurred only once in the database, all of these objects have full diagnosticity for the scene category they were found in. However, because they are rare, these objects may not be informative. Therefore, I am reporting the diagnosticity of objects with at least 10 instances in the database. The most diagnostic objects for each scene category are listed in Table 5.

**Table 5:** **The 10 most diagnostic objects for each basic-level scene category**.

**Indoor**								**Urban**				**Natural**			
**Bath**	**Bed**	**Conf.**	**Corr**	**Dine**	**Kiİn.**	**Liv.**	**Off.**	**Tall**	**City**	**Str***t*.	**High**	**Cst.**	**OpC**	**Mntn.**	**Frst.**
Bath mat	Bedspread	Microphone	Exit sign	Buffet	Cttngbrd	Coffee table	Desk mat	Skyscraper	Attic	Crosswalk	Median	Seagull	Cow	Mtn pass	Waterfall
1.00	1.00	1.00	0.90	0.94	1.00	0.94	1.00	0.97	1.00	0.89	0.89	1.00	1.00	1.00	0.92
Shower	Headboard	Podium	Arch	Silverware	Dish towel	Sofa	File	Dock	Porch	Curb	Cabin	Sand	Desert	Fog	Branch
1.00	1.00	1.00	0.89	0.84	1.00	0.78	orgnzr	0.92	1.00	0.68	0.83	0.95	1.00	0.94	0.89
							1.00								
Shwr crtn	Nightstand	Prjctn. scm	Fire alarm	Wine glass	Kettle	Ottoman	Keyboard	Antenna	Spotlight	Motorcycle	Slope	Ocean	Goose	Snow	Stick
1.00	1.00	1.00	0.84	0.78	1.00	0.72	1.00	0.90	1.00	0.66	0.65	0.93	1.00	0.89	0.88
Soap dish	Bed	Whiteboard	Blltn board	Napkin	Oven	Television	Mouse	Mast	Terrace	Van	Fence	Lighthouse	Hay	Valley	Leaves
1.00	0.99	0.73	0.52	0.70	1.00	0.61	1.00	0.89	0.94	0.55	0.64	092	bale	0.59	0.85
													1.00		
Toothbrush	Dresser	Trophy	Column	Piacemat	Pan	Armchair	Mouse	City	Arcade	Sidewelk	Bridge	Sun	River	Mountain	Land
1.00	0.93	0.67	0.47	0.55	1.00	0.60	pad	0.67	0.93	0.50	0.58	0.84	benk	0.37	0.53
							1.00						0.89		
Bath	Toy	Dsply case	Alcove	Chandelier	Stove	Fireplace	Computer	Quay	Entrance	Person	Wire	Cloud	Flowers	Cloud	Bush
0.99	0.84	0.52	0.43	0.54	1.00	0.58	0.96	0.62	0.93	0.46	0.48	0.69	0.88	0.27	0.50
Toilet	Carpet	Ashtray	Tile	China htch	Stove hd	Decoration	Monitor	Tower	Belcony	Bus	Truck	Boat	Field	Sky	Path
0.98	0.76	0.46	0.37	0.51	1.00	0.58	0.95	0.46	0.86	0.44	0.47	0.62	0.70	0.14	0.37
Twl rck	Wardrobe	Wtr bottle	Railing	Crtn rod	Dshwashr	Blanket	Filing cbnt	Garden	Garage dr	Awning	Sign	Rock	Hill	River	River
0.97	0.61	0.35	0.34	0.48	0.97	0.54	0.93	0.46	0.85	0.44	0.45	0.42	0.60	0.12	0.33
Soap	Pillow	Light swtch	Wall	Glass	Microwave	End table	Priter	Dome	Wheel	Truck	Pole	Land	Water	Ground	Rock
0.95	0.45	0.34	0.34	0.35	0.96	0.50	0.93	0.39	0.80	0.41	0.43	0.29	0.54	0.11	0.31
Tltppr	Blanket	TV stand	Floor	Candle	Utensils	TV stand	Binder	Fountain	Air cndtr	Car	Hedge	Quay	Valley	Rock	Grass
0.95	0.42	0.29	0.32	0.35	0.92	0.50	0.66	0.32	0.76	0.40	0.38	0.20	0.41	0.10	0.31

Diagnosticity is the probability of a scene being in a particular category, given the presence of a particular object. Only objects with at least ten instances are included in this table.

In addition, I examined the number of completely diagnostic objects (diagnosticity = 1) across scene categories. All objects were included in this analysis. I found that indoor scenes tended to have a higher number of completely diagnostic objects compared to outdoor scenes [25.3 vs. 11.8, *t*_(14)_ = 1.93, *p* = 0.07], although both urban and natural scene categories had the same number of completely diagnostic objects on average (11.8). Again, this is not surprising as indoor scenes had more objects overall, as well as more infrequent objects.

As noted in section Using Context for Rapid Scene Recognition, the notion of diagnosticity can be used to test hypotheses on the mechanisms of rapid scene categorization. Biederman (1981) first posited that a scene might be recognized through the recognition of a prominent, diagnostic object. How diagnostic are the largest objects in the scene? For each of the 3499 scenes, I examined the diagnosticity of the largest object in that scene for the scene's category. On average, the largest object has a diagnosticity of 0.32 for the scene category it is in (95% CI: 0.04–0.99). Thus, although knowing the identity of the largest object in the scene will allow you to guess the scene category at an above-chance level, it does not reflect the outstanding performance that human observers have with rapid scene categorization. What if you know the identity of the object nearest the center of the image? The mean diagnosticity of the center object was 0.33 (95% CI: 0.03–1.00). Although this is a little better than knowing the largest object [*t*_(6996)_ = 2.3, *p* < 0.05], it seems unlikely that human scene gist performance can be explained from recognizing the center object alone.

#### Scene-object specificity

How many scene categories contain a particular object? Here, I investigated the question by computing the number of scene categories in which each object is found. This measure is useful in the design of experiments in object and scene perception, as it allows experimenters to choose objects that are strongly tied to only one scene category (for example, to study response bias, e.g., Castelhano and Henderson, 2008) or to use objects found in a variety of scenes to de-couple object recognition from inferential effects.

**Figure 7:**
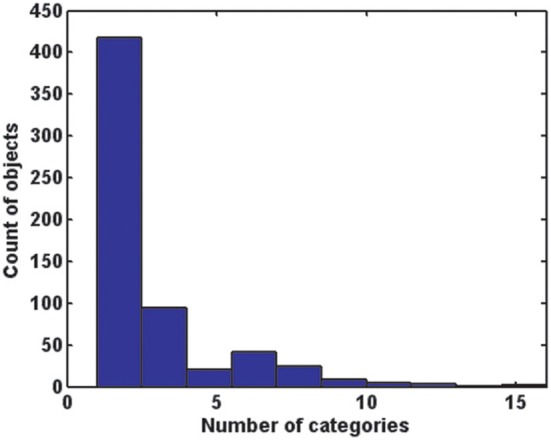
**A histogram of the number of basic-level scene categories in which each of the 617 objects is found**. The majority of objects are associated with only one or two categories.

As shown in Figure 7, the majority of objects are closely tied to one or two scene categories. The median number of scene categories containing an object was two. Forty eight percent of objects were only found in one scene category, and of these, 53% had at least two instances in the database, suggesting that this effect was not solely driven by infrequent objects. In fact, 31 of the objects found in only one scene category (5% of the total) had 10 or more instances. These are listed in Table 6. On the other hand, there was only one object present in all 16 categories (“wall”), and 19 (3% of total) were present in at least nine of the 16 categories. These are also listed in Table 6.

**Table 6:** **Objects with 10 or more database instances found in only scene category (left), and objects found in at least nine of the 16 basic-level scene categories (right)**.

**Objects in 1 category**		**Objects in 9+ categories**
Attic	Pan	Bench
Bath mat	Podium	Box
Bedspread	Porch	Chair
Cow	Projection screen	Clock
Cutting board	Seagull	Column
Desert	Shower	Decoration
Dish towel	Shower curtain	Door
File organizer	Soap dish	Lamp
Goose	Spotlight	Light
Hay bale	Stove	Person
Headboard	Stove hood	Plant
Kettle	Toothbrush	Poster
Keyboard		Rock
Microphone		Staircase
Mountain pass		Statue
Mouse		Table
Mouse pad		Tree
Nightstand		Wall
Oven		Window

#### Object pairs and groups

While the previous statistics have examined relationships between the scenes and single objects in them, it is also important to examine the relationships between multiple objects in a scene. Object co-occurrence has been shown to guide visual search in naturalistic scenes (Mack and Eckstein, 2011); interacting objects tend to be perceptually grouped (Green and Hummel, 2006); object interactions have been shown to increase activity in object-selective cortex (Kim and Biederman, 2010); and scene identity can be predicted from pairs of objects in object-selective cortex (MacEvoy and Epstein, 2011). How informative are groups of objects, and how many objects do you need to be able to predict the scene's category?

First, I examined the frequency of co-occurrence of object pairs in each basic-level scene category. The 10 most frequent object pairs for each basic-level category are shown in Table 7.

**Table 7:** **The 10 most frequent pairs of objects in each basic-level scene category**.

**Indoor**								**Urban**				**Natural**			
**Bath**	**Bed**	**Conf.**	**Corr**	**Dine**	**Kiİn.**	**Liv.**	**Îff**.	**Tall**	**City**	**Strt.**	**High**	**Cst.**	**OpC**	**Mntn.**	**Frst.**
Sink	Bed	Table	Floor	Table	Counter	Sofa	Desk	Sky	Building	Road	Road	Sky	Sky	Sky	Tree
Faucet	Pillow	Chair	Wall	Chair	Cabinet	Pillow	Chair	Building	Window	Building	Sky	Ocean	Trees	Mountain	Sky
Faucet	Bed	Ceiling	Ceiling	Window	Window	Table	Desk	Skyscraper	Door	Sky	Road	Sky	Sky	Tree	Bush
Towel	Window	Chair	Wall	Chair	Cabinet	Sofa	Monitor	Sky	Building	Building	Car	Mountain	Field	Mountain	Tree
Mirror	Bed	Light	Floor	Wtndow	Sink	Table	Monitor	Building	Door	Road	Tree	Ocean	Sky	Tree	Rock
Faucet	Lamp	Chair	Ceiling	Table	Cabinet	Pillow	Chair	Skyscraper	Window	Sky	Road	Mountain	Tree	Sky	Tree
Bath	Bed	Table	Wall	Bouquet	Window	Sofa	Window	Building	Road	Building	Tree	Sky	Sky	River	River
Faucet	Ceiling	Ceiling	Door	Chair	Counter	Lamp	Chair	Tree	Building	Car	Sky	Sand	Mountain	Mountain	Tree
Sink	Lamp	Table	Floor	Table	Faucet	Window	Keyboard	Tree	Building	Road	Sky	Ocean	Field	Snow	Grass
Towel	Pillow	Light	Door	Bouquet	Cabinet	Pillow	Monitor	Sky	Window	Car	Car	Sand	Trees	Mountain	Tree
Sink	Bed	Light	Ceiling	Ceiling	Plant	Sofa	Keyboard	Tree	Sky	Sky	Tree	Sand	Trees	Sky	Ground
Mirror	Curtain	Ceiling	Door	Chair	Cabinet	Plant	Desk	Skyscraper	Bunding	Car	Car	Rock	Tree	River	Tree
Floor	Pillow	Window	Light	Painting	Stove	Lamp	Window	Road	Sky	Building	Sign	Sky	Mountain	Rock	Bush
Faucet	Window	Chair	Wail	Chair	Cabinet	Pillow	Desk	Building	Window	Sidewalk	Road	Rock	Trees	Mountain	Sky
Towel	Bed	Window	Light	Table	Sink	Table	Keyboard	Road	Sidewalk	Sidewalk	Sign	Sand	Field	Snow	Rock
Bath	Painting	Table	Ceiling	Ceiling	Counter	Lamp	Chair	Sky	Building	Road	Sky	Mountain	Tree	Sky	River
Toilet	Ceiling	Painting	Floor	Light	Cabinet	Sofa	Book	Road	Sidewalk	Sky	Sign	Ocean	River	Sky	Mountain
Faucet	Pillow	Chair	Light	Chair	Stove	Painting	Desk	Skyscraper	Window	Sidewalk	Car	Boat	Trees	Rock	Tree
Towel	Bed	Table	Door	Light	Plant	Table	Chair	River	Road	Sidewalk	Sign	Sky	Mountain	Mountain	River
Mirror	Nightstand	Painting	Light	Table	Counter	Plant	Book	Building	Door	Car	Tree	Boat	Tree	Road	Bush

As shown in Table 7, some object pairs are functionally related (such as “faucet” and “sink” for *bathroom*), while many are not (e.g., “sky” and “building” in *skyscraper* scenes). There are 20 object pairs in this table that are listed in multiple basic-level categories. In fact, *conference rooms* and *dining rooms* share 8 of the 10 most frequent object pairs. However, of these 20 object pairs, only two are shared across superordinate-level categories (“sky” + ”building” and “tree” + ”sky”). Both of these pairs are shared across natural and urban scene categories. No object pair in this group was observed in both indoor and outdoor scenes. Therefore, although single objects may be found across all superordinate categories, pairs of objects do not share this property.

Next, I examined the 617 by 617 object co-occurrence matrix collapsed over all scene categories. Overall, the object co-occurrence matrix was sparse, with only 9% of possible object pairings having been observed. Of the observed object pairings, 8% had a co-occurrence probability of 1, indicating that these pairs of objects were always found together, and of these, 9% (*n* = 254, 0.73% of total pairings) were for objects with more than one instance in the database. Thus, requisite object pairs are relatively rare in the world, and arbitrary pairs of objects are generally not seen together.

What are the most frequent groups of *n* objects in each of the basic-level scene categories? Table 8 shows the most frequent groups of three, four, and five objects for each of the basic-level scene categories. Larger groups are not shown because many natural landscape images have fewer than 6 total objects.

**Table 8:** **The most frequent groups of three, four, and five objects found in each of the 16 basic-level scene categories**.

**Category**	**Three-objects**	**Four-objects**	**Five-objects**
Bathroom	Faucet, sink, towel	Faucet, mirror, sink, towel	Bath, faucet, mirror, sink, towel
Bedroom	Bed, pillow, window	Bed, ceiling, pillow, window	Bed, ceiling, painting, pillow, window
Conference	Ceiling, chair, table	Ceiling, chair, light, table	Ceiling, chair, light, table, window
Corridor	Ceiling, floor, wall	Ceiling, door, floor, wall	Ceiling, door, floor, light, wall
Dining room	Chair, table, window	Bouquet, chair, table, window	Bouquet, ceiling, chair, curtain, wine glass
Kitchen	Cabinet, counter, plant	Cabinet, counter, faucet, sink	Cabinet, counter, faucet, sink, window
Living room	Pillow, sofa, table	Pillow, sofa, table, window	Lamp, pillow, sofa, table, window
Office	Book, chair, desk	Chair, monitor, desk, window	Chair, monitor, desk, whiteboard, window
Tail building	Building, sky, skyscraper	Building, sky, skyscraper, tree	Building, road, sky, skyscraper, tree
Inside city	Building, door, window	Building, door, sky, window	Building, door, road, sidewalk, window
Street	Building, car, road	Building, car, road, sky	Building, car, road, sidewalk, sky
Highway	Car, road, sky	Car, road, sky, tree	Car, road, sign, sky, tree
Coast	Ocean, rock, sky	Mountain, ocean, rock, sky	Mountain, ocean, rock, sand, sky
Open country	Field, sky, tree	Field, mountain, sky, tree	Building, field, mountain, river bank, sky
Mountain	Mountain, sky, tree	Ground, mountain, sky, tree	Ground, mountain, road, sky, tree
Forest	Bush, sky, tree	Bush, river, rock, tree	Bush, river, rock, sky, tree

**Figure 8:**
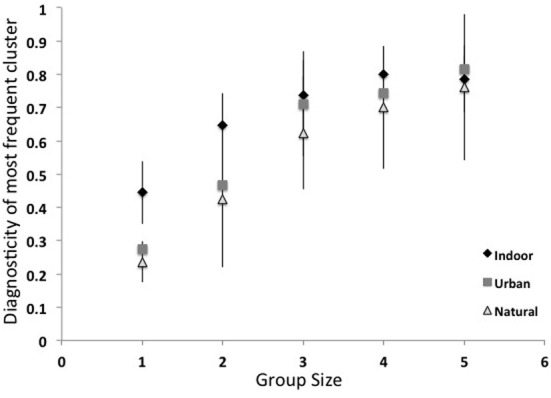
**Diagnosticity of the most frequent object groups for scene categories**.

How much information do these object groups provide about scene categories? More specifically, are these groups of multiple objects more diagnostic of a scene category than single objects? Here, I computed the diagnosticity [p(category|object)] of the most frequent groups of one to five objects. As shown in Figure 8, although the most common object in a scene category has an average diagnosticity of only 0.35, diagnosticity increases with increasing group size up to 0.78 for groups of five. The diagnosticity of object groups did not reliably differ across superordinate categories. This result gives some insight into the third path to scene recognition proposed by Biederman (1981), that scene recognition can arise through the spatial integration of a few contextually-related objects. Although this bag-of-words approach neglects the spatial relationships between objects, this analysis places a lower bound on the categorization performance that can be achieved by knowing the identities of a few objects. In section Structural Statistics, we will examine the effect of knowing coarse spatial relationships.

#### Scene combinations

How many unique combinations of objects were observed in the database? Do certain scene categories have more object combinations than others? Let us first examine the theoretical limit: if all 617 objects in the database were independent, and could occur with equal probability in all scenes, then there would be 2^617^ possible combinations of objects. Even if we examine only the possible combinations of 6 objects (the median number of unique objects in a scene from our database), this leaves us with an astounding 7.5 × 10^13^ combinations!

In contrast, I observed only 2552 unique object combinations in the 3499-scene database. In other words, 26% of scenes had the exact same combination of objects as at least one other scene in the database. However, this redundancy was not evenly distributed among the different basic-level scene categories. Ninety nine percent of indoor scenes had unique object combinations compared to only 68.6% of outdoor scenes [*t*_(14)_ = 3.71, *p* < 0.01]. Among the outdoor scenes, 85.1% of urban scenes had a unique object combination vs. 52.1% of natural scenes [*t*_(6)_ = 2.89, *p* < 0.05]. *Mountain* scenes in particular had very high redundancy in terms of unique object combinations, as only 33.7% of these scenes had a unique combination of objects.

#### Entropy

Information theory provides a formal means of expressing redundancy between objects and scene categories. If all objects in the database were independent and equally probable, then the redundancy of the database could be expressed as log2_(617)_ = 9.27 bits per object. However, object frequencies are not uniformly distributed: objects such as “chair” and “sky” are much more frequent than others such as “scaffolding” or “zebra” (section Object Diagnosticity). Relative object frequencies can be accounted for by computing the entropy of the database:

$$N=\Sigma_{p}(o)\log p(o)$$

Where *p*(*o*) refers to the observed probability of each object in the database. In this instance, taking relative frequencies into account reduces the number of bits per object needed to encode the database to 6.25. Imagine that you are trying to guess an object's identity by playing the game “20 questions.” The rules of this game stimulate that you may only ask questions whose answer is “yes” or “no.” This entropy result tells us that you would be able to correctly guess the object by asking, on average, 6 binary questions.

#### Mutual information

How much information do objects and scenes provide about one another? For example, how much evidence do you have about the category *dining room* from the presence or absence of an object such as a “chair?” To formalize this notion, I computed the mutual information between all objects and their scene categories. While diagnosticity tells us how likely an image is to belong to a particular scene category given the presence of a particular object, it does not easily tell us which objects are important, as objects occurring only once in the database are by definition completely diagnostic of that category. Mutual information measures the degree of dependence between objects and scenes and is therefore more immune to the problem of small numbers. Formally, mutual information is computed as:

I(S, O)=H(S)−H(S|O)

Where H represents entropy. More specifically, the frequency of each object O was computed for each scene category S. Thus, O and S are binary variables (O = 1 if the object is found in the image and 0 otherwise, S = 1 if the image belongs to the category and 0 otherwise). Therefore, the mutual information is:

$$\begin{array}{l} I(S,\;O)=-P(S)\text{Log}(P(S))-P(\sim S)\text{Log}(P(\sim S)+P(O)\\ \;\left(\left(P(S|O)\text{Log}(P(S|O))+P(\sim S|O)\text{Log}P(\sim \text{S}|\text{O})\right)\right)\\ \text{ +}P(\sim \text{O})(\left(P(S|\;\sim \text{O})\text{Log}(P(S|\;\sim O)\right)\\ \;+P(\sim S|\;\sim O)\text{Log}(P(\sim S|\;\sim O))) \end{array}$$

This form of mutual information is similar to that of Ullman and colleagues in computing the information between image fragments and object category (Ullman et al., 2002).

Table 9 lists the top 10 most informative objects for distinguishing between the 16 basic-level scene categories. An object can share information with a scene category either because it its presence provides strong evidence for a scene category or because its presence provides good evidence against a scene category. Thus, frequent objects that are never found in a scene category, such as “sky” in most indoor scenes or “chair” in many outdoor scenes, make the list.

**Table 9:** **The 10 objects with the highest mutual information for each of the 16 basic-level scene categories**.

**Indoor**								**Urban**				**Natural**			
**Bath**	**Bed**	**Conf.**	**Corr.**	**Dine**	**Kit'n.**	**Liv.**	**Off.**	**Tall**	**City**	**Strt.**	**High**	**Cst.**	**OpC**	**Mntn.**	**Frst.**
Towel	Bed	Chair	Wall	Chair	Counter	Sofa	Desk	Skyscrpr	Building	Road	Road	Ocean	Field	Mountain	Tree
Bath	Nghtstnd	Table	Floor	Table	Stove	Pillow	Monitor	Building	Door	Car	Car	Sand	Sky	Sky	Bush
Faucet	Pillow	Sky	Ceiling	Bouquet	Cabinet	Armchair	Keybrd	Sky	Sidewalk	Building	Sign	Sky	Window	Window	Window
Toilet	Dresser	Prjtn Scrn	Door	Sky	Pot	Coffee table	Cmptr	Ceiling	Window	Sidewalk	Median	Window	Hill	Ceiling	Colling
Sink	Lamp	Light	Sky	Buffet	Oven	Lamp	Chair	Window	Road	Person	Fence	Rock	Tree	Chair	Chair
Mirror	Curtain	Podium	Column	Plate	Sink	Ottoman	Mouse	Chair	Balcony	Sky	Sky	Ceiling	Ceiling	Table	Table
Soap	Sky	Whitebrd	Tree	Napkin	Dishwshr	Sky	Book	Table	Shp wdw	Van	Tree	Sun	Mountain	Plant	Rock
Twl rack	Painting	Building	Building	Candle	Bowl	Painting	Paper	Light	Staircase	Ceiling	Window	Boat	Chair	Light	River
Bottle	Ceiling	Tree	Exit sign	Painting	Faucet	Fireplace	Phone	Painting	Ceiling	Crosswlk	Bridge	Chair	River	Painting	Building
Sky	Carpet	Ceiling	Light	Placemat	Plate	Plant	Printer	Floor	Terrance	Chair	Ceiling	Table	Table	Snow	Light

In order to show the usefulness of objects that occur in the scene category, Table 10 lists the 10 most informative objects for each basic-level scene category, listing only those that are found in the scene category.

**Table 10:** **The 10 objects with the highest mutual information for each of the 16 basic-level categories**.

**Indoor**								**Urban**				**Natural**			
**Bath**	**Bed**	**Conf.**	**Corr.**	**Dine**	**Kit'İn**	**Liv.**	**Off.**	**Tall**	**City**	**Strt.**	**High**	**Cst.**	**OpC**	**Mntn.**	**Frst.**
Towel	Bed	Chair	Wall	Chair	Counter	Sofa	Desk	Skyscrpr	Building	Road	Road	Ocean	Field	Mountain	Tree
Bath	Nghtstnd	Table	Floor	Table	Stove	Pillow	Monitor	Building	Door	Car	Car	Sand	Sky	Sky	Bush
Faucet	Pillow	Prjtn Scrn	Ceiling	Bouquet	Cabinet	Armchair	Keybrd	Sky	Sidewalk	Building	Sign	Sky	Window	Window	Rock
Toilet	Dresser	Light	Door	Buffet	Pot	Coffee table	Cmptr	Window	Window	Sidewalk	Median	Rock	Hill	Plant	River
Sink	Lamp	Podium	Column	Plate	Oven	Lamp	Chair	Antenna	Road	Person	Fence	Sun	Tree	Snow	Building
Mirror	Curtain	Whitebrd	Tree	Napkin	Sink	Ottoman	Mouse	Door	Balcony	Sky	Sky	Boat	Mountain	Wall	Land
Soap	Painting	Tree	Exit sign	Candle	Dishwshr	Painting	Book	Tree	Shp wdw	Van	Tree	Chair	River	Door	Branch
Twl rack	Ceiling	Ceiling	Light	Painting	Bowl	Fireplace	Paper	Plant	Staircase	Crosswalk	Window	Cloud	Rock	Building	Grass
Bottle	Carpet	Projector	Arch	Placemat	Faucet	Plant	Phone	Dock	Terrace	Chair	Bridge	Mountain	Desert	Mtn pass	Road
Floor	Bedsprd	Microphone	Fire alrm	Light	Plate	Decor	Printer	Mountain	Chair	Stright	Streetlight	Road	Door	Car	Wall

*Only objects making an appearance in the scene category are listed*.

Finally, Table 11 lists the 10 objects with the highest mutual information over the entire database. These objects are the most useful for distinguishing among the 16 basic-level scene categories.

**Table 11:** **Objects with the highest mutual information for all scene categories**.

**Object name**
Sky
Building
Chair
Road
Table
Ceiling
Tree
Car
Window
Pillow

#### Scene classification with a bag of words model

Bag of words models consider a document to be represented by the list of words found within it. While visual bag of words models consider “words” output from object and feature detectors, the model we will consider here involves, literally, the list of object names within each scene. How sufficient is this representation for basic- and superordinate-level scene categorization?

**Figure 9:**
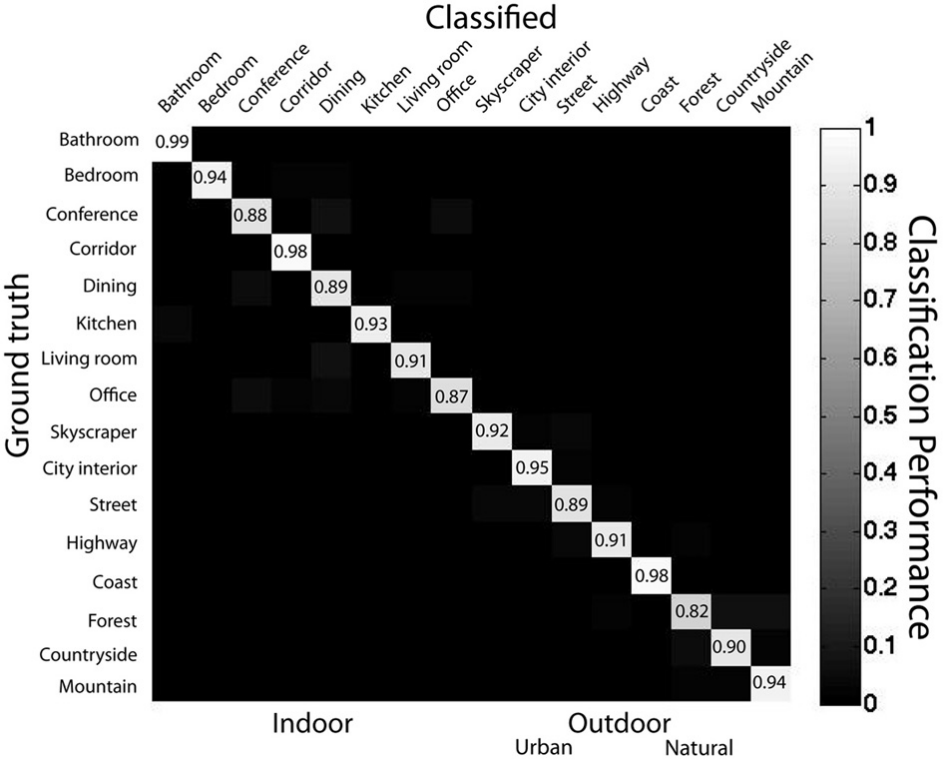
**Confusion matrix for bag-of-words model**. Data from main database using SVM with linear kernel using leave-one-out cross validation.

I also employed a linear SVM classifier trained on the raw object occurrence matrix. Here, each scene is represented as a 617-object vector where each entry represents the count of each object in that scene. The training and testing procedure was identical to that of the ensemble statistics classifier. This classifier had 98% accuracy (AUC = 0.99) at superordinate-level categorization and 92% accuracy (AUC = 0.96) at basic-level categorization; see Figure 9 for confusion matrix. A sign rank test indicated that the classifier's performance at superordinate-level classification was superior to basic-level classification performance (*Z* = 59, *p* < 0.001). There were no reliable differences in the accuracy of indoor vs. outdoor classification [*t*_(14)_ < 1], nor urban vs. natural [*t*_(6)_ < 1]. *Forest* images had the lowest categorization performance (82%), and *bathrooms* had the highest (99%). Thus, knowing all of the objects in a scene is sufficient to categorize scene images at both basic- and superordinate-levels.

**Figure 10:**
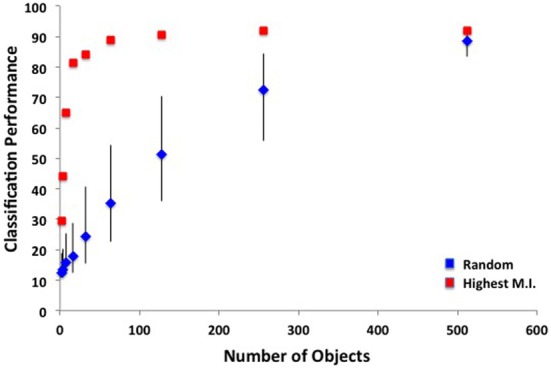
**SVM classification performance as a function of the number of objects used in a bag-of-words model**. Blue points indicate randomly sampled objects and red points indicate objects with the highest mutual information. Error bars indicate 95% confidence intervals.

To what extent is the higher performance of the bag-of-words model compared to the ensemble statistics model due to the higher dimensionality of this model? To answer this question, I ran SVM analyses on sets of six objects, either by randomly sampling from the 617 total objects, or by taking the objects with the highest overall mutual information. For sets of randomly selected objects, mean classification performance was 15.1% correct (95% CI: 13.9–16.1%), well below the 61% achieved by the same number of ensemble features. When taking the six objects with the highest overall mutual information (see Table 11), classification performance was 51.4%, only marginally worse than that of the ensemble statistic model (binomial test, *p* = 0.051). How many objects are necessary to reach ceiling performance? I ran additional SVM analyses on sets of 2–512 objects, either by randomly sampling objects or selecting objects with the highest mutual information. As shown in Figure 10, ceiling performance is reached with the 64 best objects. Therefore, although higher performance was achieved using a bag-of-words approach, this performance can be attributed to the larger dimensionality as the features contained in the ensemble statistics model contained at least as much information as a similar number of object features.

How does the performance of the bag of words model compare to the human rapid scene categorization performance reported in Kadar and Ben-Shahar (2012)? Overall sensitivity was similar between the bag of words classifier and human observers [*A*′ = 0.85 for both, *t*_(22)_ < 1]. However, the patterns of errors for the classifier and human observers were markedly dissimilar. As with the ensemble statistics classifier, error patterns were not well correlated at the basic-level (*r* = 0.04). However, error patterns at the superordinate level actually showed opposite trends from the human observers (*r* = −0.88), suggesting that the bag of words representation, although similar in performance to the human observers, is not similar to the human scene gist representation.

#### Bag of words discussion

Here I have examined statistical regularities between object identities and scene categories, ignoring the spatial relationships between these objects. The measures include object frequency, object diagnosticity, the mutual information between an object and its scene category and the number of scene categories each object is found in. At this level of analysis, the relationships that objects have to one another was also considered by examining the co-occurrence frequencies of two or more objects.

Object frequencies are not equivalent to object “consistency” as used in the visual cognition literature, which tends to be a Boolean variable (a “blender” in a *kitchen* is consistent, a “fire hydrant” in a *kitchen* is inconsistent). Here, object frequencies are continuous and range from 0 (no observed instances of this object for this scene category) to 1 (each scene in this category contains this object). This continuous scale allows the design of new experiments, allowing researchers to ask questions about the perceptual processing or memory differences that might exist for objects that are present in nearly all scene exemplars (frequency = ~1) vs. objects that are present in only about half of the exemplars (frequency = 0.5), vs. objects that are plausible but rare (frequency <0.2).

The bag of words level of analysis shows additional ways that scene categories differ. The ensemble level of analysis showed large differences between superordinate-level categories in terms of the amount of unnamed objects in scenes: indoor scenes having more than outdoor, and urban having more than natural. At this level of analysis, I found that objects strongly segregate themselves into different basic level scene categories—any given object was only found in a small number of scene categories, and when an object is found in multiple basic-level categories, these categories do not cross superordinate classes. A classifier given all object identities achieved near-ceiling performance at both superordinate- and basic-level scene classifications. Thus, knowledge of either a scene's category or an object's identity gives a great deal of information about the other, and full knowledge of all objects in a scene is sufficient for scene categorization.

Additionally, ceiling performance can be achieved with fewer objects, provided you have the “best” objects (i.e., the objects with the highest mutual information for distinguishing scene categories). Here, I demonstrated that ceiling performance could be reached with the 64 most informative objects. This is of use to those in the computer vision community who perform scene classification using hundreds of off-the-shelf object detectors (e.g., Li et al., 2010). By choosing objects that are informative, rather than frequent, these systems could be made far more efficient.

The results of the linear SVM classifier suggest that if one knows the identities of all of the objects in a scene, one will know the category of the scene. Although this has been posited as a possible route to scene understanding (Biederman, 1981), behavioral evidence suggests that human observers do not apprehend all of a scene's objects in a single glance (Fei-Fei et al., 2007; Greene and Oliva, 2009). Similarly, although the bag of words classifier had similar overall performance to human observers, it had markedly different patterns of errors, suggesting a representation different from humans. How many objects do people understand in a glance at a scene? This is a notoriously difficult problem as conceptual short term memory is relatively fragile (Potter, 1976), human observers can inflate performance through elaborate rehearsal or guessing strategies (Liu and Jiang, 2005), and observers can demonstrate sensitivity (in the form of negative priming) to objects that they cannot overtly name (VanRullen and Koch, 2003). The most stringent tests estimate that observers can only accurately report one object from a scene after a 250 ms masked display (Liu and Jiang, 2005).

Can scene recognition proceed from the recognition of just one object? When examining some plausible scenarios, such as perceiving the largest, or the most centered object, diagnosticity for the scene category is around 0.33, far below the performance of human observers in rapid scene classification. Of course, diagnosticity increases with increasing numbers of objects (section Object Pairs and Groups). However, classification performance for smaller numbers of objects, even the most informative objects, lagged behind that of the ensemble statistics model, suggesting that individual objects may not make the best features for human scene understanding and categorization.

While the bag of words level of analysis is a powerful and popular computer vision model of objects in scenes, the spatial relationships between objects and regions are also critical to scene identity. I explore this level of analysis in the next session.

### Structural statistics

The third level of object-scene relationships I will explore is aimed toward obtaining a “syntax” of visual scenes that includes the nature of the spatial relations between objects. Just as the relations between object parts are key to the object identity (e.g., a key difference between a pail and a mug is the placement of the handle, Biederman, 1987), the relations between objects may provide additional information into scene identity. The spatial layout of a scene is created in part by the relative positioning of the objects within it, and regularities in layout allow a scene to be identified under highly degraded conditions, such as under sparse contours (Biederman, 1981) or blur (Oliva and Torralba, 2007) where object identities cannot be recovered. Indeed, two of the three pathways to scene gist outlined by Biederman (1981) can come from structural relations.

Here, I will examine the locations of objects in scenes, as well as the distances between objects and the spatial distributions of the important diagnostic and informative objects. As with the other two levels of analysis, I will examine the extent to which these structural statistics can be used to classify scenes at the basic- and superordinate-levels.

#### Object position specificity

**Figure 11:**
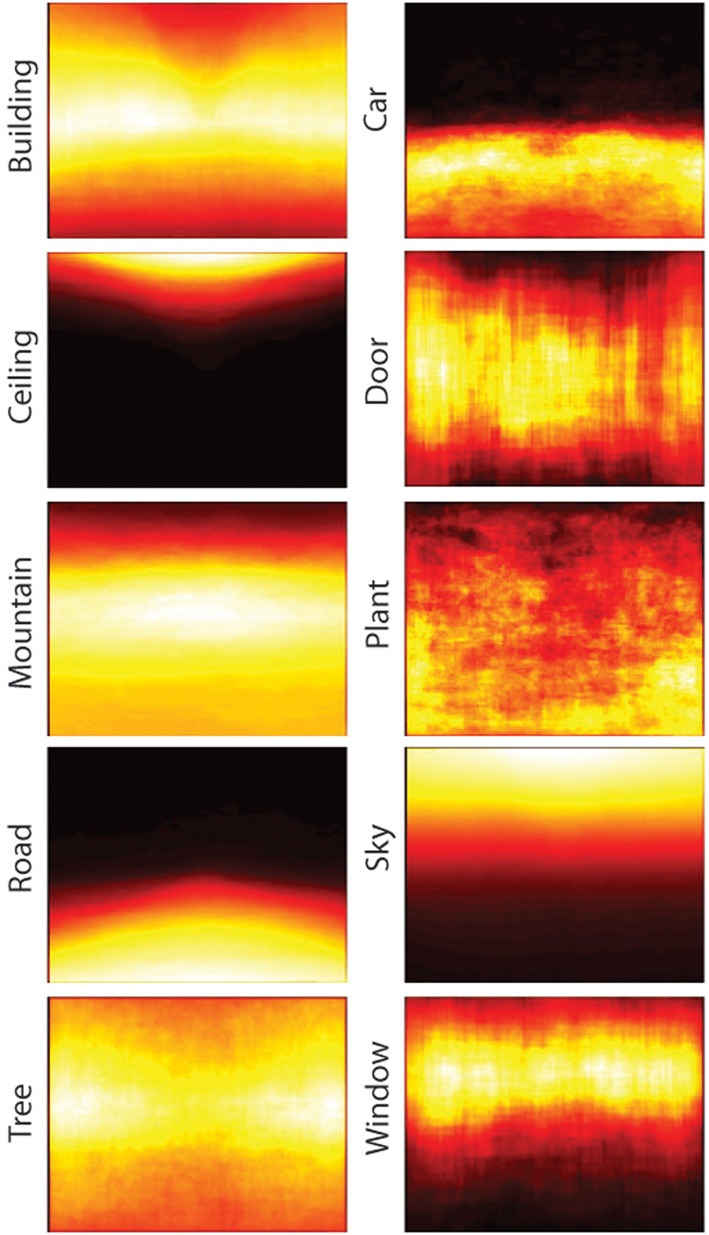
**Spatial distribution of the 10 most common objects in the database**. The pixels included in the segmentation mask for each instance of an object were summed to show the most frequent locations of objects.

One basic structural description is the position specificity of individual objects. In other words, how stereotyped are the x-y locations of the objects in the database? Figure 11 shows a heat map of the spatial locations of the 10 most common objects in the database. Some regions, such as “ceiling,” are tightly bound to a particular image location while others, such as “plant” or “building,” can be found throughout the image plane. To quantify this notion, I examined the variance in x-y position for each object center across the database as well as the position variance of objects in each of the basic-level scene categories.

Table 12 shows the 10 objects with the most position variance as well as the 10 objects with the least position variance in the database. Unsurprisingly, objects with a great deal of position specificity (low variance in x-y position) are often objects that make up the spatial boundaries of a scene (such as “carpet” and “sky”).

**Table 12:** **The 10 objects with the least position variance (most static) and with the most position variance (least static)**.

**Most static**	**Least static**
Carpet	Molding
Desert	Leaves
Ceiling	Dock
Exit sign	Dome
Bath mat	Pan
Bedspread	Basket
Ocean	Grill
Fan	Lighthouse
Bed	Calendar
Sky	Toy

*The search for these objects was constrained to objects with at least 10 instances in the database*.

For basic-level scene categories, *bedrooms* had the most position variance while *open country* scenes had the least. Overall, indoor scenes tended to have more position variance compared to outdoor scenes [*t*_(14)_ = 2.98, *p* < 0.01]. However, among the outdoor scenes, no distinct pattern emerged [*t*_(6)_ < 1].

**Figure 12:**
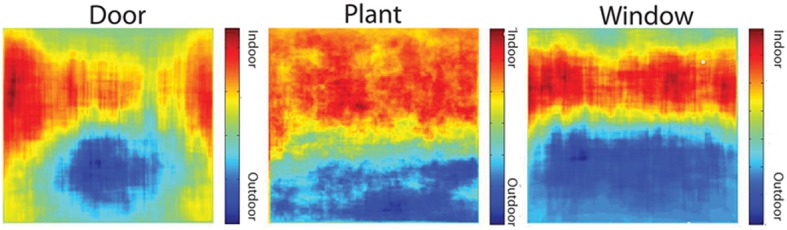
**Heat maps of the locations of doors (left), plants (center), and windows (right), conditioned on whether the object was found in an outdoor or indoor scene**. Warmer colors indicate locations that are more probable in an indoor scene while cooler colors indicate locations that are more probable for the object in an outdoor scene.

Are objects found in different locations when they are found in different scene categories? If this is the case, then position can provide diagnostic scene information. Here, I took the 17 objects that had at least 10 instances in indoor categories and at least 10 instances in outdoor scene categories (“bench,” “box,” “chair,” “clock,” “column,” “door,” “light,” “person,” “plant,” “poster,” “railing,” “sign,” “staircase,” “statue,” “trash can,” “wall,” and “window”) and examined image locations for the object when found outdoors and compared it to the locations where the object was found indoors. For each of these objects at each pixel location, I subtracted the number of instances the object was found in that location in an outdoor scene from the number of times the object was found in that location in an indoor scene. Significance was determined by Bonferroni corrected *t*-tests. Only three objects (“door,” “window,” and “plant”) had different location patterns in indoor scenes compared to outdoor scenes. Figure 12 shows that these objects are found in higher positions in the image plane when found indoors compared to where they are found outdoors. Therefore, most objects are found in similar scene locations regardless of category, so position information generally does not add additional information beyond that of object identity. Additionally, these small differences may reflect both differences in the structure of these environments (such as depth differences, as discussed in section Center of Mass), as well as differing strategies of photographers for capturing the relevant information in different environments. Our knowledge of the three-dimensional world tells us that a “door” is located in a “wall,” and just above the “floor.” Therefore, these differences reflect statistics of photographs, as well as statistics of the external world.

#### Spatial distribution of diagnostic and informative objects

Where are the most informative regions of a scene? Photographers tend to center pictures on objects of interest (Tatler et al., 2005), and objects in LabelMe tend to be labeled from the center out (Elazary and Itti, 2008). Do these centered objects have the highest diagnosticity or mutual information for their scene category?

**Figure 13:**
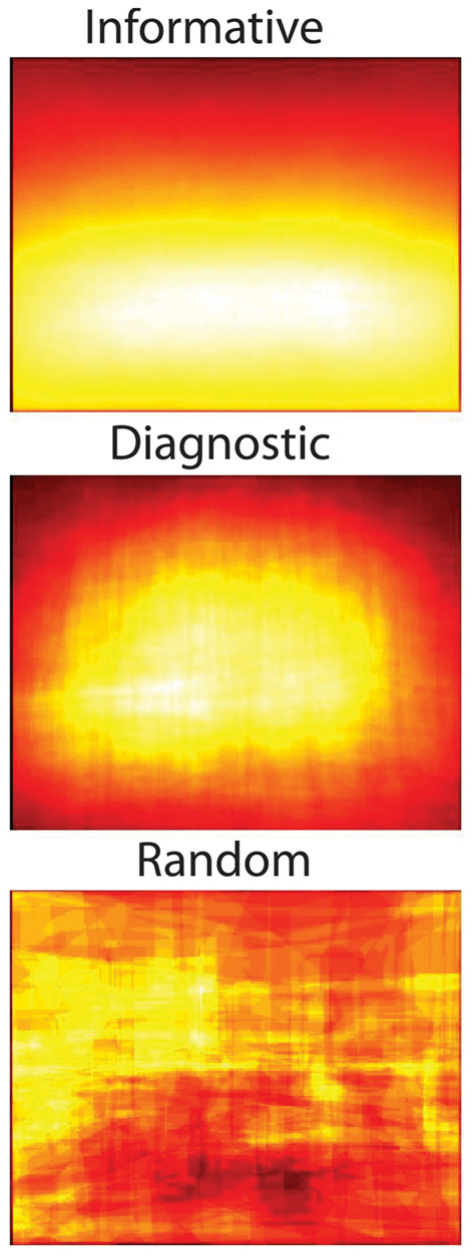
**Spatial distribution of the most informative objects for all scene categories (top), most diagnostic object for each scene category (middle) and a random object for each scene (bottom)**.

For each of the 16 scene categories, I plotted all pixels associated with that most informative object or the object with the highest diagnosticity for the scene category. As shown in Figure 13, diagnostic objects tend to be centered overall, while informative objects tend to be centered lower in the image. This is not just due to spatial regression to the center, as random selections of objects do not display this behavior.

This analysis formalizes the notion of center-bias in photographs, demonstrating that photographs tend to be centered on scene regions that contain highly diagnostic objects. Highly informative regions, on the other hand, tend to cluster near the bottom of the image. This analysis also shows key differences between the notions of mutual information and diagnosticity. Many of the most informative objects are structural or boundary elements of a scene that can coarsely distinguish between categories, but are not necessarily the most important or interesting objects in a scene (see section Mutual Information and Table 11). For example, although “carpet” is highly informative because it distinguishes between outdoor and indoor environments, it is not a terribly interesting region. As it is known that the central fixation bias of human observers persists even when important features are moved to the periphery (Tatler, 2007), this finding is unlikely to provide additional insight into human scene perception mechanisms. However, researchers in computer vision might find greater scene classification efficiency in applying object detectors from the center out rather than in a sliding window.

#### Scene classification with a structural model

How much information do object locations provide about scene categories? To answer this question, I divided all of the 3499 scene images into quadrants and computed the number of times each object was found in each quadrant for each image. Thus, compared to the bag of words model, each object is represented four times, once in each of the four quadrant locations. This matrix was fed into a linear SVM classifier with the same training and testing procedures outlined earlier. Superordinate and basic-level categorizations were assessed. Any increase in performance above the bag of words level can be taken as evidence for the utility of spatial object information for scene categorization.

Overall, this classifier had 98% accuracy (AUC = 0.99) at superordinate-level scene categorization (not significantly different from the 98% correct performance of the bag-of-words model, *Z* < 1) and 89.6% accuracy (AUC = 0.95) at basic-level categorization (significantly lower than the 92% correct from the bag-of-words model, *Z* = 3.7, *p* < 0.001). There was no reliable difference in basic-level categorization accuracy for indoor (90.8% correct) vs. outdoor (89.4% correct) scenes [*t*_(14)_ < 1], nor between urban and natural scene categories [*t*_(6)_ < 1]. Performance by category was similar to the bag of words classifier—best performance was achieved by *bathroom* (98%, tied with *corridor* and *coast*), while the classifier had the poorest performance on *open country* images (75%). These *open country* images were frequently confused with *mountains* (38.2%), *forests* (34.3%), and *highways* (14.7%).

Altogether, adding coarse spatial information to the bag of words classifier did not result in higher classification performance. This is not very surprising as the bag of words classifier was at near ceiling performance, as the majority of objects were only found in one or two scene categories (section Scene-Object Specificity), and even objects found in multiple scene categories were generally found in similar locations regardless of category (section Object Position Specificity). The lower performance for basic-level classification is likely due to an increased number of features (617 vs. 2468) with the same number of training examples.

#### Structural discussion

In this section, I have described scenes in terms of objects and their locations in the image plane. First, I described the location variability of each object, showing that objects that describe a scene's boundaries, such as “floor” or “sky” show less position variance than non-structural objects. Interestingly, most objects are found in similar locations in all scene categories. Of the objects found frequently in both outdoor and indoor scene environments, only “door,” “window,” and “plant” showed different patterns. For each of these cases, the object is found higher in the image plane in indoor scenes relative to outdoor scenes. This makes sense as the spatial enclosure of indoor scenes allows objects to be found in these locations. However, knowing an object's position in the x-y image plane does not provide much additional information over knowing its identity.

Next, I demonstrated that the center bias of photographs shows up in this database as a tendency for the most diagnostic and informative objects to be located near the center of the image. This may reflect the photographer's inherit sensitivity to object diagnosticity, and desire to convey the maximum amount of information about an environment in a single viewpoint. However, as informative objects tend to be large structural areas of a scene, diagnostic objects were more centered in the image.

Of course, both of these measures reflect statistical regularities of photographs rather than statistical regularities of the world. Although I have shown a tendency of photographers to photograph a “door” higher in the image plane in an indoor environment, we know that doors in the world are located above the “ground,” and within “walls” in all environments. Similarly, “center bias” has no meaning in the immersive, three-dimensional real world. Despite these limitations, statistics of photographs provide insight into how human observers choose to represent information from the real world when forced to choose a single view.

A linear classifier trained on the bag of words model with coarse spatial location information did not outperform the pure bag of words model, and in fact, fared a little worse in basic-level categorization. There are two reasons for this: (1) most objects are only found in one or two scene categories (section Scene-Object Specificity), so the position of these objects is not going to provide additional category-related information; and (2) of the objects that are found in several scene categories, the majority are found in similar locations regardless of category (section Object Position Specificity).

This does not mean that structural information does not contribute unique scene information, however. One limitation of measuring structural relationships on scene photographs is that we lose the three spatial dimensions that are available in the world. The third dimension would allow the disambiguation of a variety of object relationships, including containment, support and adjacency. Indeed, these types of object relations can be easily extracted using 3D models (Fisher and Hanrahan, 2010). Additionally, object pairs and groups may have spatial arrangements that are diagnostic for scene category and a more sophisticated learning approach could glean these from the data. For example, although both *dining rooms* and *conference rooms* tend to have centrally located “table” and “chairs,” and may also contain a “telephone,” the presence of telephone *on top of* the table is diagnostic of *conference room*. On the other hand, a structural description on a scene may not be a good model for human scene gist as it has been shown that human scene classification performance can be well explained as the perception of a set of unbound features (Evans and Treisman, 2005). Similarly, electrophysiological markers structural scene processing occur later than markers of semantic processing (Võ and Wolfe, 2013). Taken together, these suggest that the first scene representation may include little structural information.

As ensemble statistics had better classification performance, feature-for-feature, compared to individual objects, a structural model that coarsely localizes these types of features may prove to be more fruitful for future work.

## General discussion

In this work, I have provided a set of real world image statistics at the level of labeled objects, and assessed the utility of these measurements for scene categorization. By understanding the regularities of natural images, we can design experiments to understand how these redundancies are exploited by the human visual system to efficiently recognize environments and search for objects in those environments.

### Category information comes from different levels of analysis

I have examined scene-object relationships at three levels of analysis: the ensemble level, the bag of words level, and the structural level. Statistics measured at each level of analysis contained sufficient information to categorize scene environments into basic- and superordinate-level categories. Although we intuitively know that *kitchens* and *offices* differ in terms of the objects found in them, this work also demonstrates that scene categories differ in terms of the amount and types of “things” found in them (ensemble statistics), and to a certain degree in the spatial distribution of their objects (structural statistics).

Additionally, quantitative analysis of objects in scenes allows us to test the plausibility of hypotheses on the role of object perception in rapid scene categorization. Biederman (1981) suggested that scenes might be recognized by first recognizing a single, prominent object in the scene. In section Object Pairs and Groups, I demonstrated that knowledge of either the largest object or the most centered object was insufficient to reproduce the high classification performance of human observers. Adding additional objects increases the diagnosticity for the scene, so a path for future work will be to examine how small groups of objects might be rapidly perceived to give rise to scene gist. Classification performance using a few objects as features lagged behind classification performance of ensemble statistics, suggesting that the coarse object information provided by the ensembles was more informative about scene category than individual objects.

### Not all scene categories are created equally

Similarly, scene categories in different superordinate categories (indoor vs. outdoor, or natural vs. urban) differ markedly from one another at each level of analysis. Compared to outdoor scene categories, indoor environments have a higher object density as well as greater object variety.

The identities of the objects found in scenes also differs between superordinates, as very few objects were found in both indoor and outdoor scenes. The majority of objects in the database were found in only one or two scene categories, so knowing that an object is present in a scene provides considerable information about the scene environment. However, when considering the few objects that are found in many scene categories (such as “door,” “window,” or “trash can”), object position in the image can (but tends not to) differ by superordinate category, thus giving little additional predictive information about the scene category above that of the object identity.

Why do these indoor scene categories differ from the outdoor scene categories? One limitation of this database is that the indoor scene categories reflect small-scale indoor environments in the home and workplace. Perhaps larger indoor environments such as *department store* or *warehouse* would show patterns more similar to the outdoor environments, as larger environments mean that more objects will be too small to individually label, leading to a smaller number of measured objects.

Interestingly, categorization accuracy for all superordinate-level categories was found to be similar for each of the classifiers considered here. This was unexpected, as indoor scene categorization is often considered to be a harder problem than outdoor scene categorization (Quattoni and Torralba, 2009). This result suggests that machine vision systems performing indoor scene categorization can be improved in at least two ways: first, the use of “objectness” detectors (Alexe et al., 2012) could be employed to understand object density and other ensemble statistics that are somewhat diagnostic of scene categories, and second, to use object detectors for the objects that provide the most mutual information for distinguishing scene categories.

### Are all object types created equally?

Throughout this paper, I have treated each annotated label equally for the purposes of statistical analysis. “Sky” is just as much of an object as “book” in the database, even though it is not tangible and has no clear boundaries in the world. Although defining what counts as an object is a notoriously difficult problem (for a review see Feldman, 2003), one might want to consider sub-types of objects. For example, one might distinguish between object labels that refer to count nouns vs. mass nouns (Burge, 1972; Adelson, 2001; Huntley-Fenner et al., 2002; Prasada et al., 2002). Count nouns are labeled objects that are discrete and countable (“mug,” “building,” “car,” “book”) while mass nouns are regions with no fixed units or boundaries (“field,” “water,” “smoke,” “sky”). This distinction appears to be a fundamental difference in object representation that is present from a very early age (Huntley-Fenner et al., 2002). Alternatively, some of the annotated labels reflect background or boundary elements of a scene, such as “ground,” “sky,” “wall,” or “ceiling.” As a well-accepted definition of a visual scene includes the lawful arrangement of objects on a background (Henderson and Hollingworth, 1999), it is possible that these labeled regions have a different perceptual status than other labels such as “bowl” or “book.” Indeed, objects that make up scene boundaries have the highest mutual information for distinguishing between scene categories (see Table 11). However, a glance at the labels in Appendix B will convince the reader that it is very easy to find unclear cases.

### Generalizability and database bias

How generalizable are these findings? In other words, how much do they say about the distribution of objects in the world, and how much do they say about the idiosyncrasies of this particular database? Although the eight-category database from Oliva and Torralba (2001) used in the main database is a standard scene classification set in computer vision, more modern work has criticized this database for relying too heavily on the Corel Stock Photo collection (Torralba and Efros, 2011), which may represent only over-stylized representations of scenes. Similarly, the indoor images largely consist of highly idealized environments from real estate websites. Do these generalize to more everyday environments? In order to address this question, I have computed all statistics on a separate auxiliary database, and I have shown the similarities and differences between the two datasets whenever possible in Appendix D. Assuming that the bias in these datasets is independent, their degree of overlap reflects the generalizability of these statistics (Torralba and Efros, 2011; Khosla et al., 2012). This assumption is likely to be optimistic, however, as both datasets are part of the larger set of scenes that people find remarkable enough to photograph and share on the web in the first place.

The two datasets examined in this work showed considerable but not perfect overlap. It is likely that the noted differences between natural landscapes and indoor environments are robust to dataset bias, but perhaps not the differences between urban and indoor scenes. The auxiliary set showed that the differences in these superordinates is driven primarily by natural landscape images, as this database contained very complex urban environments whose images had object density similar to indoor environments. Both datasets showed remarkable overlap in object frequency and mutual information, making these measures generally useful for the design of new experiments on object-scene context. Similarly, the measured entropy was very similar between the two datasets, suggesting that this statistic is robust to any dataset bias. The specificity of objects to a particular scene category was also observed in both sets. However, other measurements should be taken with more caution. The main dataset showed more redundancy (scenes having the same combination of objects) than the auxiliary set, and this manifested itself in higher classifier performance across the board. Appendix D contains more details on the specific differences in the findings between the two data sets.

Future investigations will continue to validate the generalizability of these data via comparison to other annotated databases such as SUn (Xiao et al., 2010), or through modeling the bias directly (Khosla et al., 2012). Separately, one can see how these statistics match the intuitions of human observers, although observers' intuitions should not necessarily be counted as ground truth, because we are insensitive to statistical base rates in some domains (Tversky and Kahneman, 1974).

### The utility of object context statistics

Although it is generally recognized that lawful contextual relationships facilitate scene and object recognition, work in this area has been limited because these contextual relationships have not been fully characterized and quantified. Previous work has characterized contextual relationships as merely being the intuitive plausibility of an object for a given scene environment. Many of the scene-object pairs in these experiments include informative but rare objects, such as a “moose” in a *forest*. Although a moose is more likely to be found in a forest when compared to other types of environments, the vast majority of *forest* images will not include a “moose.” By measuring object frequency, diagnosticity and mutual information, experimenters will be able to determine the perceptual and memory consequences of these relationships individually. Furthermore, current experiments treat contextual relationships as binary—an object is either contextually related to an environment or it is not. However, the statistics measured here are continuous, allowing for more subtle questions to be asked.

More broadly, it has been argued that we cannot yet perform well-controlled studies on natural scene images because it is too difficult to understand or control the stimuli (Rust and Movshon, 2005). The results presented here take a necessary step toward this goal by characterizing complex scene stimuli in terms of quantified object-scene relationships. At all levels of analysis, real-world scene images show remarkable redundancy that can be utilized by the brain to represent the world efficiently. Therefore, measuring these statistics allows us to better understand and control our stimuli and to move forward into more real-world vision research.

### Conflict of interest statement

The author declares that the research was conducted in the absence of any commercial or financial relationships that could be construed as a potential conflict of interest.

## Footnotes

^1^Upon publication, this database and Matlab structures containing statistical data will be publically available for download on the author's website.

## Appendix A: raw labels (SIC)

<blank>, “?object,” abinet, adding machine, advert, aerial, aerial building, air conditining, air conditioner, air conditioning, air conditioning occluded, air filter, air vent, airco, airplane, alarm, alarm clock, alarmclock, alcove, all, American flag, animal, animal seal, animals, antique bureau, apple, apples, arcade, arcade occluded, arch, arches, archway, arearug, arid ground, arm chair, armchair, armchair back, armchair part, amchair top, armoir, art, art piece, art?, artichokes, ashtray ash tray, attic, automan, avenue, awning, awning crop, awning occluded, awning tidy up, back of chair, back wall, backpack, badge, bag, balcany occluded, balcony, balcony crop, balcony occluded, bale, balustrade, bandstand, banister, bank, bar, barbeque, barrier occluded, base board, basket, basket food, basket fruits, basket of fruit, basket of magazines, basket of towels, basket table, basket of brushes, baskets, bath, bath faucet, bath mat, bath tub, bath robe, bathtub, bathtub plug, beach, beam, bed, bed part, bed posts, bed skirt, bedskirt, bedspread, bell, bell pepper, bell tower, bench, bench occluded, bib, biclist occluded, bicycle, bicycle occluded, bicyclist, bicyclists, bidet, bidet faucet, bilding, bin, bin occluded, binder binders, bird, bird figurine, bird occluded, birdcade, blackboard, blancket, blanket, blender, blind, blinds, block, block of cheese, blocks, bluildings, boad, board, board games, boarder, boat, boat crop, boat cropped, boat decoration, boat occluded, boats, book, book case, book shelf, book shelves, bookcase, books, books on shelf, bookshelf, bookshelves, boooks, boot, boots, border, bottl;e, bottle, bottle top, bottles, bottom bunk, bottom of chair, bottom of chairs, bouldings, bouldings occluded, bouquet, bouquet flowers, bowel, bowl, bowl of apples, bowl of fruit, bowl of popcorn, bowl of strawberries, bowl of vegitables, bowl with food, bowl with fruit, bowl with vegis, bowls, box, box contants, box occluded, box?, boxes, braided garlic, branch, branches, branchs, brand name, brand name crop, brand name occluded, bread, bread tray, brick fireplace, brick wall, bridge, bridge crop, bridge handrail, bridge occluded, briefcase, broom, brushes, brush, brushes, bucket, buffet, buggy, buiding, buiding occluded, buidings occluded, buildig occluded, buildin, buildin occluded, building, building aerial, building crop, building façade, building occluded, building occluded, building skyscraper, buildingl occluded, buildings, buildings crop, buildings occluded, buildings occludeds, buildingsoccluded, buildins, buildins occluded, buildintgs occluded, buildling, buildlings occluded, bulletin board, buoy, bus, bus stop, bus occluded, bus stop, bus stop occluded, bushes, business card, bus occluded, cabin, cabin occluded, cabinet, cabinet door, cabinet dresser, cabinets, cabinets, cabintet shelf, cake, cake dish, cake stand, calander, calendar, calender, can, canal water, candel, candle, candle holder, candle stand, candle stick, candles, canister, canister with brushes, canister with utencils, canisters, cannon, canoe, canopy, cans, captus, captus crop, car, car crop, car side, car az90deg, car crop, car frontal, car frontal occluded, car occluded, car ocluded, car rear, car rear az90deg, car rear occluded, car rear side, car rear side crop, car rear side crop, car side, car side az0deg, car side az180deg, car side crop, car side occlude, car side occluded, car side rear, car side rear crop, car sides, car_back, car_front, car_left, car_right, car_top_back, car_top_fornt, car_top_front, card, carpet, carpet floor, carrots, cars, cars occluded, cars side, cars side crop, cars side occluded, cars sides, cart, case, cassettes, castle, cat, cd, cd's, cdoor, cds, ceiing light, ceililng, ceiling, ceiling fan, ceiling lamp, ceiling lamps, ceiling light, ceiling molding, ceiling tile, ceiling vent, ceiling lamp, ceilng light, celing, cell phone, centerpiece, centra reservation, central reservation, ceramic rooster, certain, certificate, chair, chair back, chair bottom, chair crop, chair leg, chair legs, chair occluded, chair part, chair seat, chair top, chair wheel, chairs, chaise lounge, chandalier, chandelier, chandelier, chandlier, changing table, channel, char, cheese, chess board, chessboard, chest, chiar, child walking, chimney, china, china cabinet, china hutch, china plate, cigarettes, city, cliff, clip board, clock, clock occluded, closet, closets, cloth, clothes, clothes hamper, cloths, cloud, clouds, coaster, coat, coat rack, coatrack, coffee maker, coffee pot, coffee table, column, column, column occluded, columns, comforter, computer, computer monitor, computer screen, computer tower, computers, conference table, cooler, copier, cords, couch, counter, counter top, counter, cove, covered balcony, covered balcony occluded, cow, cows, cpboard, crane, crane occluded, crib, cross, crosswalk, cubbord, cubby, cubical, cubicle, culumn, cup, cup and saucer, cup holders, cup with flower, cupbard, cupboard, cups, curb, curtain, curtain rod, curtains, cushion, cutting board, cutting board island, cutting board with vegitables, cyclist, dam, decoration, decorations, decorative ball, decorative balls, decorative boat, decorative bowl, decorative box, decorative fish, decorative kimono, decorative mask, decorative mirror frame, decorative molding, decorative object, decorative objects, decorative pillow, decorative plat, decorative plate, decorative pots, decorative tree, decorative urn, decorative wall hanging, decoratvie plate, deoderant, derer ground, desert, desert field, desert ground, desk, desk calendar, desk calender, desk divider, desk lamp, desk mat, desk organizer, desk separator, desks, digital watch, dining table, disc, dish, dish rack, dish towel, dish towels, dishes, dishrack, dishwaher, dishwasher, disks, display case, display stand, dock, dog, dog crop, doily, doll, dolphin, dome, dome crop, dome occluded, door, door crop, door entrance, door frame, door knob, door mat, door occluded, door_, doorframe, doorpart, doors, doorway, double door, double door crop, double door occluded, double window occluded, drape, drapes, drawer, drawer nob, drawers, drawyers, dresser, dressing screen, dressor, dried flowers, dried plant, drinking fountain, driver, dry earase board, dry earaser, dry erase board, dryer, dune, eagle, egg crate, eggplant, eilling lamp, electric mixer, electrical outlet, elevator door, elf, embankment, emplem, enclave, end board, end table, entertainment center, entrance, entrance occluded, entry, entry-phone, entryway, envelope, envelopes, equipement, esplanade, estate, exit sign, external driver, external drivers, eye, falg, fall branch, fan, faucet, faucit, faucst, fax machine, fence, fence crop, fence occluded, fences crops, fern, ferns, field, field desert, field flowers, field grass, figurine, figurines, file, file box, file cabinet, file cabinets, file organizer, files, filing cabinet, fining cabinet_, filing cabinets, fire, fire alarm, fire escape, fire extinguisher, fire hydrant, fire place, fire sprinkler, firehose, fireplace, fireplace screen, firewood, fish tank, flag, fip flop, fllor, flock, floor, floor carpet, floor marble, floor, carpet; floor_, floral centerpiece, flower, flower arrangement, flower in vase, flower pot, flowers, flowers in pot, flowers in vase, flute, flyer, flyers, fog banck, fog bank, folder, folders, food, food on plate, foot board, footboard, footbridge, footrest, forest, forest mountain, fork, forks, fountain, fountain crop, fountain occluded, fp screen, frame, framed mirror, framed picture, frozen over river, fruit, fruit bowl, fruit in bowl, fruit plate, fruit stand, fruits, fruits bowl, fruits plate, frying pan, funicular (railway), garage, garage door, garage door occluded, garages, garden, garland, garlic, garment bag, gas pump, gas station, gate occluded, glass, glass bowl, glass cupboard, glass door, glass doors, glass piece, glass shelf, glass table, glass wall, glasses, globe, goblet, goblets, goose, goose occluded, gooses, grandfather clock, grapes, grass, grass field, green field, green peppers, greenhouse, grill, grille, grille occluded, groound, groun grass, ground grass crop, ground snow-covered, groung grass, hair, hair brush, hall, hand rail, hand towel, hand vaccum, handle, handrail, hanger, hanging bird, hanging fish, hanging hat, hanging instrument, hanging lamp, hanging light, hanging monitor, hanging pan, hanging pans, hanging patches, hanging pitcher, hanging plant, hanging plate, hanging pot, hanging pot rack, hanging rack, hanging rug, hanging toy, hanging utencils, hanging utensils, hanging utentcil rack, hanging wall flower, harbor, hat, hay bale, head board, head stand, headboard, headphones, hearth, heater, hedge, highway, hill, hill urban, hills, hold back, hood, horse, hot pad, house, house occluded, house crop, house in ruins, house occluded, houses, houses crop, houses crops, houses occluded, houses occludeds, human, hung out, hutch, hydrant, ice bucket, idol, ilver ware, image frame, in box, industry, inset ceiling light, inset ceiling lights, instrument, intercom, ipod, iron, island, isle, jar, jars, jet, jetski, jetty, jewelry box, joist, joists, jug, junk, kettle, key board, key board shelf, key pad, keyboard, kiosk, kite, Kleenex, Kleenex box, knife, knife holder, knife holdrer, knife set, knives, knobs, ladder, ladle, lake, lake water, laminating machine, lamp, lamp part, lamp shade, lamps, lampshade, land, lap top, laptop, large bowl, large window, lava, leaf, leaves, leaves tree, ledge, lemon, letter bin, lettuce, lichen, light, light fixture, light switch, lighter, lighthose, lighthouse, lights, lignthouse, line persons, liquor bottle, litter bin, litter bin crop, little bear, lodge, loft, logs, lotion, lots of chairs, lower cabinets, machine, machines, magazine, magazine holder, magazine rack, magazines, magnifier, magnifying glass, mailbox, mailboxes, make up case, man, manhole, mantle, map, markers, massage table, massager, mast, mat, matt, mattress, message board, message board_, metal door, metal door occluded, metal shutters crop, microphone, microwave, might stand table, mill, mini blinds, mirror, mirror extender, misc., mixer, molding, monitor, monitor stand, monitor_display, monitors, monitpr, monkey, monkey occluded, monolith, monolith crop, monument, moon, motorbikde occluded, motorbike, motorbike crop, motorbike occluded, motorbike side, motorbike side crop, motorbike side occluded, motorbikes side, motorclyclist, motorcyclist, motorcyclist crop, mountain, mountain pass, mountains, mountainside, mountan, mouse, mouse pad, mouse stand, moutain, mouth, movie screen, muffin, mug, mugs, napkin, napkin in glass, napkins, neck pillow, news stand, news-stand, newspaper, night stand, night stand cabinet, night stand dresser, night stand part, night stand table, nightstand, notebook, notebooks, nothing, notice, nozzle, nutcracker, obelisk, object, object, objetc, objectds, objects, occluded, occluded sky, ocean, ocean water, oject, onion, orange, ottamon, ottoman, ounter top, outlet, overhead projector, pad, paht, pail, painting, paintings, paitings, pallet, palm tree, palm pilot, palm tree, palm tree crop, palm tree cropped palm tree occlude, palm tree occluded, palm tree trunk, palm trees, palms trees, pan, pane, pane crop, pane occluded, panel, panelling, panels, pants, paper, paper filer, paper roll, paper sheet, paper towel, paper towel holder, paper towels, paper weight, papers, park, parking lot, parking meter, parking place, parquing door, parquing meter, marquing meter occluded, pass window, passway, pasta, path, pbject, pear, pears, pedestal, pedestel, pedestrian street, pen, pen box, pen holde, pen holder, pen set, pencil, pencil cup, pencils, pens, people, people riding, people sitting, people walking, pepper mill, pepper shaker, perkalator, person standing occluded, person, person standing, person walking, person boy, person boy standing, person crop, person cyclist, person in line, person man back crop, person man sitting, person man standing, person man walking, person man walking occluded, person occluded, person riding, person setanding, person sitting, person sitting cropped, person sitting occluded, person skiing, person skiing crop, person skiing occluded, person stading, person stangig, person standing crop, person standing kid, person standing occluded, person sweping, person swiming, person swimming, person waling, person walkin, person walking, person walking crop, person walking occluded, person walking ocluded, person wallking, person wise, person wise back, person wise back occluded, person wise backoccluded, person wise crop, person wise in profile, person wise occluded, person woman, person woman sitting, person woman standing, person woman walking, person working, persons rowing, persons standing, persons walking, peson standing, peson walking, pesrson walking, pestal and mortar, pet bowl, phone, photocopier, piano, picture, picture frame, pictures, pie, pig statue, pillar, pillow, pillows, pipe, pitcher, placard, place mat, place matt, placemat, plain, plams trees, plan pot, plank, plank occluded, plank, plant, plant box, plant box occluded, plant crop, plant fern, plant grapevine, plant in stand, plant in vase, plant occluded, plant pot, plant pot occluded, plant stand, plant tree, plants, plants fern, plants ferns, plaque, plate, plate and bowl, plate of fruit, plates, platform, platter, plug, plumbing, pneumatic tire, podium, pole, pole crop, pole occluded, poles, pond, porch, porch occluded, portfolio, portico, post it note, post it notes, poster, poster board, poster crop, poster occluded, posters pole, pot, pot holder, pot plant, pot plant crop, pot plant occluded, pots, potted flowers, potted plant, power cord, pperson driving occluded, precipice, printer, prjection screen, prjector, projection screen, projector, prson standing, prson walking, puddle, puffy object, pulley, purse, pylon, pylon occluded, qall, quay, quilt, rack, rack of fruit, radiator, radio, radio alarm clock, rail, railing, railings, raill, railroad track, railway, rainbow, range hood, recycling bins, red light, ree, reflection, refridgerator, refrigerator, refuge, remote, remote control, revolving doors, rig, river, river bed, river side, river water, riverside, rives water, road, road highway, road traffic, roads, roamn shade, roasting pan, robe, rock, rock cropped, rocke wall, rocks, rocky ground, rocky hill, rocky mountain, rocky mountains, rocky moutain, rocky plain, roks, rolling pin, roman shade, roof, roof occluded, room label, rooster, rooster figurine, rootes, round table, row of chairs, row of desks, rubber duckie, rug, ruin, runner, s, sack, sailboat, sailing boat, sair railing, salt shaker, sand, sand beach, sands, caofa, satellite dish, sauce pot, saucer and bowl, scaffolding, scaffolding crop, scale, scanner, sconce, screen, scrub brush, scrubland, sculpture, sculpture crop, sculpture occluded, sculpyure, sea, sea water, sea beach, sea water, seagull, seashell, serving tray, sewer, shade, shades, shadow, shampoo, shedders, sheep, shelf, shell-bowl of popuri, shelves, ship, ship occluded, shop window, shop window crop, shop window occluded, shower, shower curtain, shower curtain rod, shower door, shower faucet, shower head, shower nozzle, shrub, shrub cropped, shrubs, shrubs crop, shrubs occluded, shudder, shutter, side, side rail, side tabel, side table, side walk, sideboard, sidetable, sidewalk, sidewalk café, sig, sign, sign crop, sign occluded, signes, signs, siky, silver ware, silverware, sing, sink, sink faucet, site office, sk, skier, skier crop, skly, sky, sky light, sky occluded, sky sunset, skyscraper occluded, skyscrape, skyscraper, skyscraper building, askyscraper crop, skyscraper occluded, skyscraperoccluded, skyscrapers, skyscrapers occluded, skyscrapr occluded, skyscrapre occluded, skyscrpaer occluded, skyscrper occluded, skysscraper occluded, slated wooden panel, sled, sleepers, sleeping robe, sliding door, sliding door crop, sliding glass door, slipper, slippers, slope, sloped ceiling, small bowl, small plate, small rug, small table, small table part, small vase, smoke alarm, smoke detector, snow, snow covered, snow covered ground, snow covered mountain pass, snow covered plain, snow covered road, snow covered valley, snow land, snowly mountain, snowy covered field, snowy ground, snowy hill, snowy mountain, snowy mountain pass, snowy plain, snowy road, snowy trees, soap, soap bars, soap bottle, soap box, soap dish, soap dispenser, soap holder, soap on a rope, soaps, socket, sofa, sofa bed, sofa part, soil, soldier, sonwy mountain, sown field, space heater, spatulas, speacker, speaker, spice jar, spice rack, sping, sponge, spoon, spotlight, spray bottle, sprinkler, ssnowy mountain, stacj of papers, stack of books, stack of papers, stack of plates, staicase, stained-glass window, stainless steel back splash, stainless steel splash guard, stainless steel wall, stair board, stair railing, staircase, staircase occluded, stairs, stairway, stake, stand, stand occluded, stands, stapler, starage rack, starfish, station, statue, streetlight, step, steps, stero, stick, sticker, sticks, stone, stone ball, stone ball crop, stone occluded, stone vase with flowers, stones, stones wall, stool, storage box, storage rack, stov, stove, stove gaurd, stove hood, stove nob, stove sheild, stove top, streelight, street, street light, street lighting, street market, streetcar, streetlamp, streetlight, streetlight crop, streetlight occluded, streetlights, streetlilght, string, stump of tree, subway, suitcase, sun, sun occluded, sunflower field, sunflowers field, sunflowers field, sunset, supermarket, supplies, support beam, tabel occluded, table, table cloth, table lamp, table leg, table occluded, table part, table runner, table top, table with tablecloth, tablecloth runner, tableland, tables, tank, tanker occluded, tapestry, tea cup and saucer, tea kettle, tea pot, teapot, teddy bear, telephone, telephone booth, telephone box, telephone box occluded, television, television cabinet, television case, television screen, television stand, tence, terotauuko, terrace, terrace occluded, text, thermos, thermostat, tile, tile ?, tiled wall, tissue, tissue box, tissues, toast, toaster, toaster oven, toilet, toilet brush, toilet brush stand, toilet paper, toilet paper holder, toilet paper roll, toll gate, tomatoes, toolbox, tooth brush holder, tooth brushes, toothbruch holder, toothbrush, toothbrush in cup, toothbrush in jar, toothbrushes, toothpaste, tootlbrush, top bunk, top chair, top of chair, top wall, torch, towel, towel hanger, towel rack, towels, tower, tower crop, tower occluded, town, toy, toys, tp roll, track, track lighting, tractor, traffic lights, traffic light, traffic light frontal, traffic light side, traffic lights, traffic lights crop, traffic sign, trafficlights, trash, trash can, trash compactor, tray, tray table, tray with supplies, trays, tree, tree crop, tree cropped, tree cut down, tree in pot, tree leaves, tree occluded, tree top, tree trunk, tree trunk crop, tree trunk cropped, tree trunk fallen, tree trunk occluded, tree trunks, trees, trees cropped, trees occluded, trees occludeds, trees top, trophy, trres, truck, truck crop, truck frontal, truck frontal occluded, truck occluded, truck rear, truck rear occluded, truck side, truck side occluded, trucks occluded, truk, truk occluded, truk side occluded, trumpet, trunk, trunk tree fall, tub, tube, tuck occluded, tumbledown building, tunnel, tv, tv stand, typewriter, umbrella occluded, umbrellas, undergroth, undergrowth, urban plain, urban valley, urbanization, urinal, urn, utencil, utencil holder, utencil rack, utensil, utensils, valance, valley, van, van crop, van frontal, van occluded, van rear, van rear side, van side, van side crop, van side occluded, vanity, varehouse, vase, vase with flowers, vase with leaves, vase with plant, vases, vault, VCR, vegetable, vegetables, vegetation, vegitable, vegitables, vegitables in bowl, vegitables on a plate, vehicles, vent, vents, verge, vertical blinds, verticle blinds, viewpoint, vineyard, volcano, votive, wakk, wal, wall, wall boarder, wall border, wall clock, wall hang, wall hanging, wall lamp, wall light, wall mount, wall occluded, wall outlet, wall outlets, wall stitch, walls, wardrobe, wardrobe part, wash cloth, washcloths, washing machine, waste basket, water, water bottle, water bottles, water cooler, water fall, water fountain, water ocean, water pond, water river, water sea, waterfall, watering can, watermelon, wathervane, wave splash, waves, web cam, webcam, wheat field, wheelbarrow, wheelchair, wheels, white board, white out, whiteboard, whte board, widnow ledge, wind chime, windex, window, window ceiling, window crop, window frame, window ledge, window occluded, window pane, window seat, window shade, window shades, window shop, window shudders, window shutter, window sill, windows, windwo, wine bottle, wine cupboard, wine glas, wine glass, wine glasses, wine rack, wineglasses, wineglass, wineglasses, wire, wire rack, woman, woman walking, wood, wood beam, wood post, wooden ship, workstation, x, xx, xxx, zebra, zebra crossing, zebras.

## Appendix B: final region labels

Adding machine, advert, air conditioner, air filter, airplane, alarmclock, alcove, antenna, apple, arcade, arch, armchair, artichokes, ashtray, attic, awning, backpack, bag, balcony, bandstand, barbecue, base board, basket, bath, bath mat, bath plug, bathrobe, beach, beam, bed, bed posts, bedskirt, bedspread, bell, bell pepper, bench, bicycle, bidet, binder, bird, birdcage, blanket, blender, block, board games, boat, book, bookshelf, boot, bottle, bouquet, bowl, box, braided garlic, branch, bread, bridge, briefcase, broom, bucket, buffalo, buffet, buggy, building, bulletin board, buoy, bus, bus stop, bush, business cards, cabin, cabinet, cactus, cake, cake dish, cake stand, calendar, can, canal water, candle, candle holder, canister, cannon, canoe, canopy, car, card, carpet, carrots, case, cassettes, castle, cat, cave, cds, ceiling, ceiling fan, ceiling tile, cell telephone, centerpiece, certificate, chair, chaise lounge, chandelier, changing table, chart, cheese, chessboard, chest, chimney, china, china hutch, cigarettes, city, cliff, clip board, clock, closet, cloth, clothes, clothes hamper, cloud, coaster, coat, coat rack, coffee maker, coffee table, column, computer, computer monitor, cooler, counter, cow, crane, crib, cross, crosswalk, cubicle, cup, curb, curtain, curtain rod, cutting board, dam, decoration, deer, deodorant, desert, desk, desk divider, desk mat, desk organizer, dish, dish towel, dishrack, dishwasher, display case, dock, dog, doily, doll, dolphin, dome, door, door mat, drawer, dresser, dressing screen, drinking fountain, dryer, dune, eagle, egg crate, eggplant, electric mixer, electrical outlet, elevator door, emblem, end table, entertainment center, entrance, envelope, equipment, eraser, esplanade, estate, exit sign, external hard drive, fan, faucet, fax machine, fence, fern, field, file, file organizer, filing cabinet, fire, fire alarm, fire escape, fire extinguisher, fire hydrant, fire sprinkler, firehose, fireplace, fireplace screen, firewood, fish tank, flag, flock, floor, flower pot, flowers, fog, food, footboard, footrest, forest, fork, fountain, frame, fruit, fruit bowl, fruit stand, frying pan, garage, garage door, garden, garden, garland, garlic, garment bag, gas pump, gas station, gate, glass, glasses, globe, goblet, goose, grandfather clock, grapes, grass, greenhouse, grill, ground, hair brush, hall, hanger, harbor, hat, hay bale, headboard, headphones, hearth, heater, hedge, hill, horse, house, instrument, intercom, ipod, iron, island, jar, jetski, jetty, jewelry box, jug, kettle, keyboard, kiosk, kite, knife, knife set, knob, ladder, ladle, lake, laminating machine, lamp, lamp shade, land, laptop, lava, leaves, ledge, lemon, lettuce, lichen, light, light switch, lighter, lighthouse, lodge, loft, lotion, machine, magazine, magazine rack, magnifying glass, mailbox, make up case, manhole, mantle, map, markers, massage table, massager, mast, mat, mattress, median, microphone, microwave, mirror, mirror extender, molding, monitor stand, monkey, monolith, monument, moon, motorcycle, mountain, mountain pass, mouse, mouse pad, muffin, mug, napkin, news stand, newspaper, nightstand, notebook, nozzle, nutcracker, obelisk, object, ocean, onion, orange, ottoman, oven, overhead projector, painting, pallet, palm pilot, pan, panel, pants, paper, paper, roll, paper towels, paper towels holder, paper weight, park, parking lot, parking meter, parking place, pasta, path, pear, pedestal, pen, pen holder, pen set, pencil, pencil cup, pepper shaker, person, pestle and mortar, pet bowl, photocopier, piano, picture, pie, pillow, pipe, pitcher, placard, placemat, plank, plant, plant pot, plaque, plate, plateau, platform, platter, plumbing, podium, pole, pond, porch, portfolio, post it note, poster, pot, pot holder, pot rack, power cord, printer, projection screen, projector, puddle, pulley, purse, pylon, quay, quilt, rack, radiator, radio, railing, railroad track, railway, rainbow, recycling bins, reflection, refrigerator, remote control, river, river bank, road, rock, rolling pin, roof, room label, roots, rubber duckie, rug, ruin, salt shaker, sand satellite dish, scaffolding, scale, scanner, sconce, scrub brush, seagull, sealion, seashell, sewer, shadow, shampoo, sheep, shelf, shelves, shoe, shop window, shower, shower curtain, shower door, shower head, shutters, sideboard, sidewalk, sidewalk café, sign, silverware, sink, site office, sky, sky light, skyscraper, sled, sliding door, slippers, slope, smoke detector, snow, soap, soap dish, soap dispenser, soap on a rope, sofa, soil, space heater, spatulas, speaker, splash guard, sponge, spoon, spotlight, spray bottle, sprinkler, stained glass window, staircase, stake, stand, stapler, starfish, station, statue, step, stereo, stick, sticker, stool, stove, stove hood, stove top, street market, streetcar, streetlight, string, stump of tree, subway, suitcase, sun, supermarket, table, table runner, tablecloth, tank, tapestry, teapot, teddy bear, telephone, telephone booth, television, television stand, terrace, thermos, thermostat, tile, tire, tissue, toast, toaster, toilet, toilet brush, toilet brush stand, toilet paper, toilet paper holder, toll gate, tomatoes, toolbox, toothbrush, toothbrush holder, toothpaste, torch, towel, towel rack, tower, toy, tractor, traffic light, trash, trash can, trash compactor, tray, tray table, tree, trophy, truck, trumpet, trunk, tunnel, typewriter, umbrella, urinal, urn, utensils, utensils rack, vacuum, valley, van, vanity, vase, vault, VCR, vegetable, vent, verge, vineyard, volcano, wall, wall hanging, wall mount, wardrobe, warehouse, washing machine, watch, water, water bottle, water buffalo, water cooler, water fountain, waterfall, watering can, water cooler, water fountain, waterfall, watering can, watermelon, wave, weathervane, webcam, wheel, wheelbarrow, wheelchair, white out, whiteboard, wind chime, windex, windmill, window, window frame, window ledge, window seat, window shade, window sill, wine bottle, wine glass, wine rack, wire, wood, wood post, zebra.

## Appendix C: labels that were deleted (SIC)

Bottom of chair, box contants, chair leg, chair wheel, eye, hold back, junk, lamp part, misc., mouth, nothing, occluded, precipice, side, sping, supplies, table leg, terotauuko, viewpoint, xx, xxx.

## Appendix D: auxiliary data set

- Object density
  Unique object density for the auxiliary set was highly correlated with the main data set (*r* = 0.82). While natural landscapes in the auxiliary set had fewer objects when compared to urban scenes [*t*_(6)_ = 3.46, *p* < 0.05], there was no reliable difference in object density in this database between indoor and outdoor environments [*t*_(14)_ < 1], as urban scene images in this database were more complex than urban images in the main set.
- Classification using ensemble statistics
  For the auxiliary set, images could be classified at the superordinate level with 85% accuracy (AUC = 0.86). Likewise, basic-level categorization could be done on this data set with 33% accuracy (AUC = 0.61). Although the categorization performance is significantly above the chance level of 6.3%, it is lower than the 61% achieved by the main database (*Z* = 13.8, *p* < 0.0001). To what extent is this lower performance due to the smaller size of the database? I sampled 5000 sets of 1220 images from the main database and tested classification performance on these sets, and found a median basic-level classification accuracy of 51% (95% confidence interval: 48–54%). Thus, although database size explains some of the performance difference, the higher classification performance on the main dataset may reflect homogeneity in the images that might not be reflected in the real world. This does not diminish from the main point of this analysis: that simple ensemble statistics of objects in scenes provides above-chance information about scene categories.
- Ten most frequent objects in auxiliary set
  (In descending order of frequency). Wall, sky, floor, window, tree, road, building, lamp, door, table.
- Overlap between frequent objects in main database vs. auxiliary database
  How did the object frequencies measured in the main database compare to the object frequencies in the auxiliary set? For each basic-level scene category, I computed the overlap between the 10 most frequent objects in both databases. Overall, there was 69% overlap, ranging from 50% (*conference room*) to 90% (*bathroom*).
- Object diagnosticity
  To compare object diagnosticity between the main and auxiliary datasets, I computed the overlap of the 10 most diagnostic objects in both sets. 34% of the 10 most diagnostic objects in the main set were in the 10 most diagnostic objects in the auxiliary set.
  The 10 most diagnostic objects in each basic-level category: (In descending order of diagnosticity). *Bathroom*: sink, shower, shower door, soap, toilet lid, toilet paper, towel rack, toilet, towel, bath. *Bedroom*: bed, nightstand, dresser, pillow, painting, telephone, closet, fan, clock, picture. *Conference room*: board, object, laptop, bottle, ground, bench, bicycle, speaker, chair, table. *Corridor*: entryway, light, light switch, bench, door handle, door, poster, ceiling, trash can, umbrella. *Dining room*: plate, dish, flower, vase, fireplace, tray, radiator, armchair, knife, bowl. *Kitchen*: cutlery, dish towel, stove hood, oven, coffee maker, stove, dishwasher, refrigerator, microwave, blender. *Living room*: sofa, fireplace, magazine, television stand, television, pillow, carpet, armchair, furniture, vase. *Office*: mousepad, keyboard, computer monitor, computer, desk, mug, mouse, pen, speaker, can. *Tallbuilding*: skyscraper, river, bus, building, statue, roof, fire hydrant, streetlight, pole, chimney. *City*: umbrella, house, balcony, parking meter, street, motorcycle, traffic light, wheel, van, awning. *Street*: hedge, wire, manhole, headlight, shutters, windshield, tail light, truck, van, license plate. *Highway*: bridge, median, tail light, fence, grass, road, cloud, truck, car, skyscraper. *Coast*: beach, ocean, sand, boat, cliff, rock, water, sky, cloud, mountain. *Open country*: field, hill, grass, cloud, bush, bison, house, sky, mountain, snow. *Mountain*: bison, snow, mountain, forest, hill, cloud, water, house, rock, sky. *Forest*: path, tree trunk, forest, foliage, river, snow, ground, tree, rock, grass.
  The smaller degree of overlap for diagnosticity, compared to frequency (section Object Frequency) or mutual information (section Mutual Information) is likely due to the fact that the auxiliary set is much smaller, so there are fewer objects with at least 10 instances in this dataset.
- Scene-object specificity
  Forty six percent of objects were found in only one category, and of these, 43% had more than one instance in the database. A total of 19 objects (3.6%) were found in nine or more scene categories. Comparing these 19 objects to the 19 objects from the main database found in nine or more categories, 13 objects were found in both. These are: “box,” “chair,” “clock,” “door,” “lamp,” “light,” “person,” “plant,” “poster,” “table,” “tree,” “wall,” “window.”
- Number of unique combinations
  In the 1220 image auxiliary set, 1108 images had unique object combinations (9% redundancy).
- Entropy
  The entropy of the auxiliary set was 6.15 bits per object.
- Mutual information
  Fifty four percent of the 10 most informative objects for each scene category were shared between the main database and the auxiliary set, and six of the 10 objects with the highest overall mutual information for all scene categories were shared between the two data sets.
- Classification using bag of words model
  Ninety seven percent accuracy (AUC = 0.97) at superordinate-level categorization (not significantly different from main set performance: *Z* = 1.2, *p* = 0.23) and 80.2% basic-level categorization accuracy (AUC=0.88). This was significantly lower performance compared to the main dataset (*Z* = 14, *p* < 0.001). As the two data sets contained different objects, it is not possible to train on one and test on the other as in the previous section.
- Classification using structural model
  The auxiliary set achieved 77.5% correct at basic-level categorization (significantly lower than main dataset: *Z* = 13, *p* < 0.001) and 97% correct at superordinate-level categorization (similar to performance on main data set: *Z* < 1).
